# Connectivity between nidopallium caudolateral and visual pathways in color perception of zebra finches

**DOI:** 10.1038/s41598-020-76542-z

**Published:** 2020-11-09

**Authors:** Yi-Tse Hsiao, Ta-Ching Chen, Pin-Huan Yu, Ding-Siang Huang, Fung-Rong Hu, Cheng-Ming Chuong, Fang-Chia Chang

**Affiliations:** 1 Department of Veterinary Medicine, School of Veterinary Medicine, National Taiwan University, Taipei, Taiwan; 2 Department of Ophthalmology, College of Medicine, National Taiwan University, Taipei, Taiwan; 3 Research Center for Developmental Biology and Regenerative Medicine, National Taiwan University, Taipei, Taiwan; 4 Institute of Veterinary Clinical Science, School of Veterinary Medicine, National Taiwan University, Taipei, Taiwan; 5 Department of Pathology, University of Southern California, Los Angeles, CA USA; 6 Graduate Institute of Brain and Mind Sciences, College of Medicine, National Taiwan University, Taipei, Taiwan; 7 Graduate Institute of Acupuncture Science, College of Chinese Medicine, China Medical University, Taichung, Taiwan; 8 Department of Medicine, College of Medicine, China Medical University, Taichung, Taiwan

**Keywords:** Cognitive neuroscience, Computational neuroscience, Neural circuits, Visual system

## Abstract

Researchers demonstrated an elegant ability for red discrimination in zebra finches. It is interested to understand whether red activates exhibit much stronger response than other colors in neural network levels. To reveal the question, local field potentials (LFPs) was recorded and analyzed in two visual pathways, the thalamofugal and the tectofugal pathways, of zebra finches. Human studies demonstrate visual associated telencephalons communicate with higher order brain areas such as prefrontal cortex. The present study determined whether a comparable transmission occurs in zebra finches. Telencephalic regions of the thalamofugal (the visual Wulst) and the tectofugal pathway (the entopallium) with their higher order telencephalon, nidopallium caudolateral (NCL) were simultaneously recorded. LFPs of relay nuclei (the nucleus rotundus, ROT) of tectofugal pathway were also acquired. We demonstrated that LFP powers in the tectofugal pathway were higher than those in the thalamofugal pathway when illuminating blue lights. In addition, the LFP synchronization was stronger between the entopallium and NCL. LFPs also revealed a higher Granger causality from the direction of entopallium to NCL and from ROT to entopallium. These results suggest that zebra finches’ tectofugal pathway predominately processing color information from ROT to NCL, relayed by entopallium, and blue could trigger the strongest response.

## Introduction

Like humans, birds heavily rely on color vison to gather information and perception^1^. Color discrimination affects their food seeking and mate choosing abilities^2,3^. The central visual pathways in brain exhibit similar organizations between mammals and birds. In humans, there are two major pathways—the geniculocortical and extra-geniculocortical pathways, which process visual information. Similarly, the thalamofugal and the tectofugal pathways in birds are respectively corresponding to the geniculocortical and extra-geniculocortical pathways^1,4^. Unlike humans, who depend mainly on the geniculocortical pathway to process visual information^5,6^, birds with laterally placed eyes have the most prominent tectofugal pathway (corresponding to extra-geniculocortical pathway in mammals)^7,8^. Lesions of this pathway cause deficits on color discrimination tasks^9^.

However, with estimating approximately 10,000 species of birds, merely the eye position (laterally placed eyes or medially placed eyes) resulted in profound differences in how their brains process visual information^1^. Even in the species with similar eye positions, there still are species-specific variations for avian brains. The zebra finches’ (*Taeniopygia guttata*) retina contains an extra type of cone cells, ultraviolet cones, in addition to the short-wavelength (S)-cones, medium-wavelength (M)-cones, and long-wavelength (L)-cones^10^, suggesting that ‘blue’, ‘red’, and ‘green’ play important roles for zebra finches. Researchers found that the color of seed feeders attracted different species of garden birds, implying their different color preferences^11^. Red is the best known for its function than other colors for zebra finches. The capability of red color discrimination seems critical for both male and female zebra finches to decide on their mates^3,12–14^. Recently, researchers also demonstrated an elegant ability of red discrimination in zebra finches^15^. Most of the literatures focus on the correlation between red and social behaviors^3,12–15^. But, it is also interesting to investigate the neural network level of whether red activates much strong responses than other colors, and whether the zebra finches are indeed sensitive to other colors than red. A report demonstrates that zebra finches have green, blue, and UV light-dependent magnetic compass, suggesting that blue light is critical for their navigation^16^. The present study recorded local field potentials (LFPs) in the zebra finches’ brain regions and tried to elucidates the brain response when they were stimulated by various colors. Next, we introduced the brain regions that were recorded in our experiments.

The visual information from the retina transmits to both the optic tectum and the geniculatis lateralis pars dorsalis (GLd) of the thalamus in birds. The neurons in the optic tectum project to the nucleus rotundus (ROT), which then send the signals to the entopallium (ENTO). This optic tectum-ROT-ENTO projection pathway is referred to as the tectofugal pathway^17,18^. Hodos et al. conducted a series of lesion experiments to determine the functions of ROT and ENTO. Deconstruction of ROT or ENTO cause deficits in the pattern discrimination^19,20^, visual intensity^19,20^, and color discrimination^4,9^. Reports further elucidate that the tectofugal pathway processes motion signal, color perception, luminance changes, and in-depth vision signals, and is also sensitive to looming (approaching) objects^4,21^. It seems that the tectofugal pathway is in charge of some simple visual signals^4^. On the other hand, the signal from GLd transmits to the visual Wulst (VW), a telencephalic region that is comparable with mammalian visual cortex^22^. This pathway is called the thalamofugal pathway. The thalamofugal pathway is important for birds, which relies on binocular vision to hunt preys (e.g., owl) in long distance and requires precise perception of three-dimensional stimuli^23,24^. However, the functions of VW in owl are not comparable with zebra finches^25,26^, since zebra finches are laterally eyed bird that results in only marginal binocular interaction for both of their eyes^27^. Seed or fruit-eating birds use frontal binocular vision only when they peck foods from where is close to their beaks. Zebra finches are granivores (seed predators) with laterally placed eyes and mostly depend on the monocular vision^25^. A previous report showed that male zebra finches prefer to use right eye to observe females and show off their flanks during the early ritual of courtship^28^. The color preference for zebra finches is still an unsolved question and most studies employed behavioral tests to determine. In this study, we elucidated what kinds of color spectra trigger the largest brain responses in aforementioned visual pathways. For this purpose, we implanted electrodes into the nuclei (ROT, ENTO and VW) relayed in the tectofugal and thalamofugal pathways in the left hemisphere, then shined a series of colors to the right eye, and acquired the LFPs from these nuclei. The above mentioned ENTO and VW are parts of telencephalon and are also belong to the tectofugal pathway and thalamofugal pathway, respectively^4,8^. Moreover, the avian telencephalon divides into two parts, the Wulst (in the rostral region of the brain) and dorsal ventricular ridge (in the caudal region of the brain)^4,8^. In addition, dorsal ventricular ridge comprises a sub-region called nidopallium, and ENTO transmits visual information to the nidopallium for further cognitive process^8^. Primate or human studies reveal that the visual associated telencephalon communicates with higher order of brain areas, such as the prefrontal cortex^29–31^. In pigeons, the nidopallium caudolateral (NCL) is equivalent to the prefrontal cortex in mammals^32–34^. NCL in pigeon executes functions such as planning and decision-making^33,34^. In chickens, NCL also involve in imprinting behavior^35^. Recently, a study indicates that the location and trajectory of NCL are species-specific; the patterns of dopaminergic innervations are strikingly different between pigeons/chickens and zebra finches^32^. These findings imply that the zebra finch’s NCL may modulate different functions than those of pigeons/chickens. The function of NCL in zebra finch is not well understood. It may involve in regulation of arousal or courtship behavior^36^. The present study also explored the potential role of NCL in zebra finches when processing color information. Because the tectofugal pathway possesses the ability in color information^4,9^, we hypothesized that telencephalons of visual pathway communicate with higher order brain areas in zebra finches and ENTO has stronger communication with NCL than VW, which is similar to humans^29–31^, when stimulated by colors. We initially expected a prominent response in ENTO when zebra finches see colors. We also hypothesized that zebra finches are more sensitive to red because of the mate-selection^3,12–14^, but blue and green may be also important for the navigation purpose^16^.

In addition to revealing the optimal projection pathway which responds to certain color(s), we ask if this pathway communicates with a higher order of brain region for cognitive processing when zebra finches perceive the particular color(s). To address this question, we implanted additional electrode to the NCL, which resembles mammalian prefrontal cortex, to acquire LFPs. LFPs are electrical potentials generated by neurons in a local brain region and can determine the brain activities from the network to systemic level^37–41^. By analyzing the amplitude (or power) of LFPs from a single area, the synchronization between connected brain areas, and the directional connectivity between paired brain areas, we can pave the way for neurophysiological investigations of color information processing in the aspects of activities (relevant to power), communication (relevant to synchronization), and the leading direction (relevant to directional connectivity) between (or within) the tectofugal pathway and the NCL or between the thalamofugal pathway and NCL. In addition, the degree of sensitivity for different colors in brain level was also revealed in the experiments.

## Results

### Tectofugal pathway mainly mediates brain responses to colors

**Figure 1:**
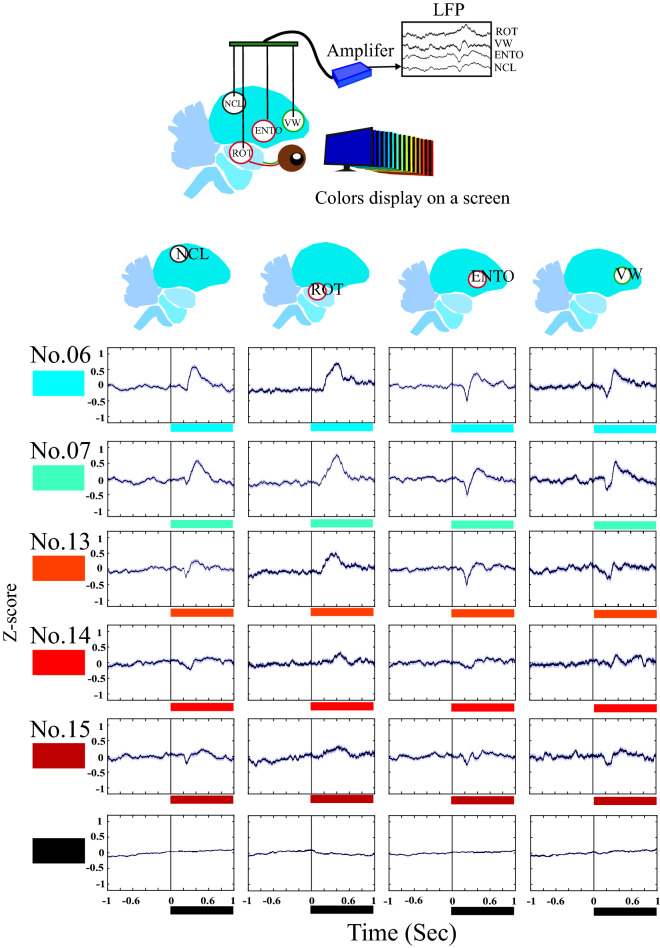
The averaged LFP traces in the brain regions after stimulated by 15 colors with black color as baseline between each color. The top illustration displays the experimental protocols. The stimulation trials were the unit for analysis. The LFP amplitudes of y-axis were Z-scored and depicted as the means ± SEMs. The zero at the x-axis is the stimulation time point. The evoked potentials were emerged about 500 ms after the stimuli and LFPs possessed their own waveforms in each of these four regions. In this figure we represent 5 color stimuli, the detailed LFP traces after stimulated by 15 colors have been shown in Fig. S1.

**Figure 2:**
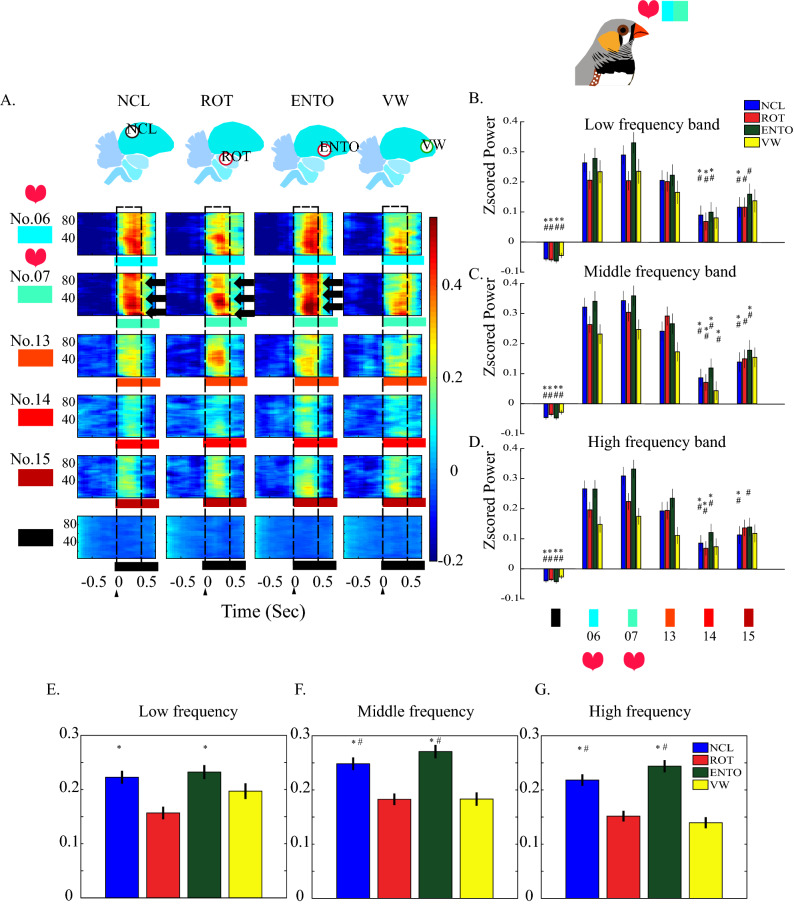
The averaged spectrograms in the brain regions after stimulated by 15 colors with black color as baseline between each color. Here we selected results from only red stimuli and blue stimuli for comparing the brain responses to red and blue. The stimulation trial as the unit to be analyzed. (See Fig. S2 for the complete 15-color-stimuli spectrograms.) (**A**) The Z-scored spectrograms of red and blue stimuli. Frequencies (Hz) have shown on the y-axis and stimulation time point is marked as zero at the x-axis. Dashed line box marks the time between 0 and 500 ms. The magnitude of power is color-coded and the power scales are plotted under each column. Although the animals were recorded under anesthesia, the color No. 7 evoked the strongest power and the high energy revealed in different frequency bands (depicted by arrows). (**B**,**C**,**D**) are the mean of powers between 0 and 500 ms (from the dashed line boxes in (A)) of low, middle, high frequency bands, respectively. The values were depicted as means ± SEMs. * denotes the *p* value < 0.05 when compared to color No. 6; # denotes the *p* value < 0.05 when compared to color No.7. (One-way repeated measurement ANOVA, then Bonferroni post hoc comparison). (**E**,**F**,**G**) display the mean of powers between 0 and 500 ms without differentiating the 15-color-stimuli. The values were depicted as means ± SEMs. *Denotes the *p* value < 0.05 when compared to the ROT; #denotes the *p* value < 0.05 when compared to the VW (one-way ANOVA, then Bonferroni post hoc comparison).

**Figure 3:**
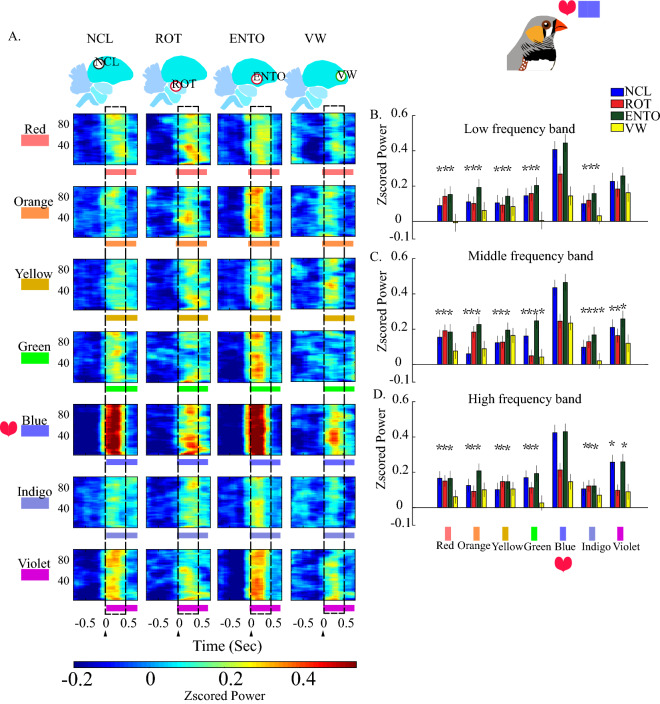
The averaged spectrograms in the brain regions after stimulated by rainbow colors with black color as baseline between each color. (**A**) The Z-scored spectrograms of baseline and rainbow color stimuli. Frequencies (Hz) have shown on the y-axis and stimulation time point is marked as zero at the x-axis. The magnitude of power is color-coded and the power scales are plotted under each column. We further confirmed the results from the 15-color-stimulation by using the rainbow colors with similar radiation intensity. (**B**,**C**,**D**) are averaged powers within the time period (0–500 ms) of low, middle, and high frequency bands. The values were depicted as means ± SEMs. *Denotes the *p* value < 0.05 when compared to blue. (one-way repeated measurement ANOVA, then Bonferroni post hoc comparison; stimulation trials as the unit to be analyzed).

We are interested in the brain structures to interpret the meaning of color stimulation. We recorded the LFPs from the relay nuclei of the thalamofugal and tectofugal pathways in brains. To study the effects of different colors on activating thalamofugal and tectofugal pathways, we used red, green, and blue (RGB) codes from 0 to 1 with 0.25 increment to create 15 colors (for details, Table S1; see "Materials and Methods" section). Fifteen colors were flashed to the subjects’ right eye (Figs. 1, S1–S3) and simultaneously recorded the LFPs at the relay nuclei of the thalamofugal and tectofugal pathways, owing to the fact that male zebra finches usually use right eye to observe females during courtship^28^. We averaged the LFPs across the same color stimuli with the same background color (black background color (Figs. 1 and S1)). Although the subjects were recorded under general anesthesia, the evoked potentials still emerged between 0 and 500 ms after color flashing when black background was used as baseline (Fig. 1). The LFP traces of evoked potentials were dissimilar among the four recording targets. As displayed in Fig. 1, the evoked potentials in NCL were slower and had indistinct negative potentials (the valley of NCL peaked after 250 ms) when compared with those of ENTO (the valley peaked at approximately 250 ms), VW (the valley peaked at approximately 250 ms), and ROT (nearly no valley occurred). Therefore, the LFPs demonstrated no contamination from volume conduction. Volume conduction is an electric current transmitting between nearby brain areas and usually shows no phase delay in LFPs between sources and the nearby areas, as indicated that the recorded waveforms are similar between different brain areas and may mask the actual LFP signals. The different shape and response time of evoked potentials among regions in our LFP recordings suggest that volume conduction did not cause artificial synchronization and the acquired LFPs were real signals. Therefore, it is worth to further analyze the functional connectivity. It has been demonstrated that gamma oscillations are triggered by vision through the tectofugal pathway in pigeons^42^; therefore, we investigated the changes of LFP powers in different spectra after receiving the different 15-color stimuli before further analyzing the functional connectivity. The LFP spectrograms stimulated by 15 different colors depicted that the strongest responses were elicited after receiving blue to green lights (Figs. 2A, S2, color No. 6: RGB color (0, 1, 1), and color No. 7: RGB color (0.25, 1, 0.75)). However, if the white background color was used as the baseline, no distinct response was found among these color stimuli (Fig. S3). The spectrograms of color stimuli displayed a predominant power between middle and high frequencies from the onset of the trigger to half a second after the trigger (Fig. 2A dashed line box). The powers between the middle and high frequency bands were increased (as the arrows indicated in Fig. 2A); therefore, we referred to rat’s frequency bands and divided the responsive spectrograms into three frequency bands: the low frequency (< 20 Hz, including the delta, theta and alpha waves), the middle frequency (20–60 Hz, mainly containing slow gamma waves), and the high frequency (60–100 Hz, mainly containing fast gamma waves)^43^. Bar graphs of average powers across 0–500 ms after stimuli depicted that the subjects have low responses to the reds (colors No.14 or No.15) when comparing to colors No. 6 or No. 7 in the ROT and ENTO (Fig. 2B–D analyzed by one-way repeated measures ANOVA, Bonferroni-adjusted significance tests for pairwise comparisons; see supplementary Table S2, for detailed means ± SEMs, F values, *p* values, and corrected *p* values). NCL also showed strong response to blue (Fig. 2B–D, analyzed by one-way repeated measures ANOVA, Bonferroni-adjusted significance tests for pairwise comparisons; see supplementary Table S2, for detailed means ± SEMs, F values, *p* values, and corrected *p* values). These results suggested that subjects were sensitive to the illumination of blue (color No. 6, 7) colors when comparing with red (color No. 14, 15) colors. This phenomenon was only observed in VW in middle frequency when flashed with No. 14 color (Fig. 2B–D, analyzed by one-way repeated measures ANOVA; see supplementary Table S2 for detailed means ± SEMs, F values, *p* values, and corrected *p* values). The overall Z-scored powers in ENTO are significantly higher than those of ROT and VW (Fig. 2E–G: analyzed by one-way ANOVA compared between total of 15 colors among 4 areas, Bonferroni-adjusted significance tests for pairwise comparisons. See supplementary Table S3A for detailed means ± SEMs, F values, *p* values, and corrected *p* values). The overall Z-scored power in NCL are also strong (Fig. 2E–G, supplementary Table S3A). These results suggested that the NCL-ROT pathway plays the major role in processing color information. We further created 7 rainbow colors with the same radiation power (Table S1), since the previous 15 colors created by the color codes did not control the radiation power. After adjusting these rainbow colors to an identical radiation power, the colors were a little bit pale (Fig. 3A; color index on the left; also see "Materials and Methods" section, Table S1). The radiation-power-controlled rainbow colors still triggered evoked potentials (Fig. S4, LFP traces). In addition, the rainbow colors still generated strongest power in ENTO and significantly stronger than VW (Fig. S5A, analyzed by one-way repeated measure ANOVA compared between the total rainbow colors among 4 areas, Bonferroni-adjusted significance tests for pairwise comparisons. See supplementary Table S3C for detailed means ± SEMs, F values, *p* values, and corrected *p* values). Since there were some missing values from VW recording because of the broken recording wires and one-way repeated measure ANOVA excludes the missing trials, we analyzed the statistical differences by one-way ANOVA. The statistical analysis still demonstrates significant higher power in ENTO than VW (see supplementary Table S3B for detail statistic values). The blue colors triggered the highest potentials, especially in ENTO and NCL (Figs. 3B–D, and S5B, analyzed by one-way repeated measures ANOVA, Bonferroni-adjusted significance tests for pairwise comparisons; see supplementary Tables S4 and S3D for detailed means ± SEMs, F values, *p* values, and corrected *p* values). The powers in ROT and VW are still relatively low (Fig. 3A–D), which is consistent with the findings using 15-color stimuli. We were interested in knowing whether blue trigged highest response in ENTO than VW, therefore, we analyzed z-scored power after the blue stimulation. The data elucidate a significant stronger power in ENTO than VW (Fig. S5B, One-way repeated ANOVA, see supplementary Table S3D for detailed means ± SEMs, F values, *p* values, and corrected *p* values).

### Phase synchronization between the NCL and the tectofugal pathway is stronger

**Figure 4:**
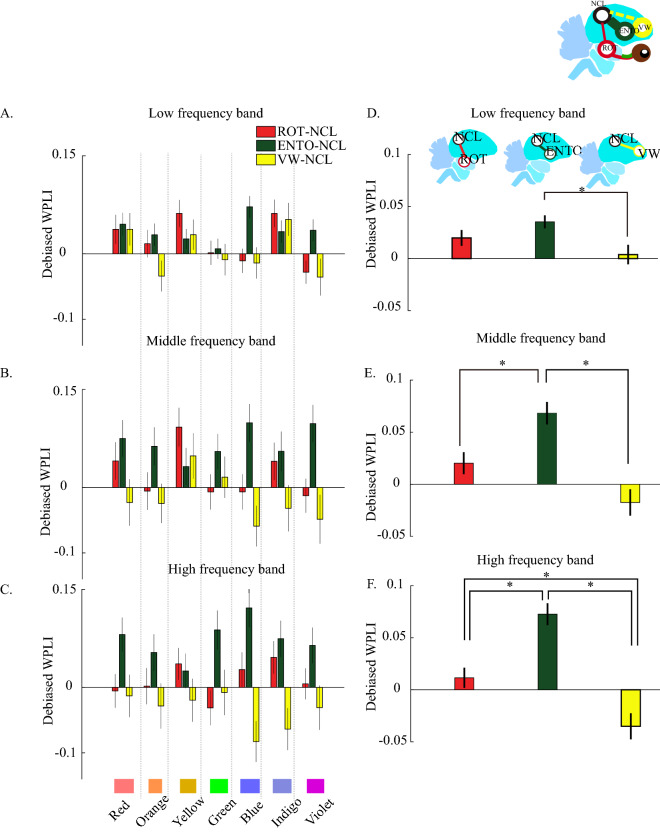
Phase synchronization between the NCL and other 3 brain regions after stimulated by rainbow colors with black color as baseline between each color. (**A**,**B**,**C**) are the means of WPLIs within the time period (0–500 ms) for the 3 frequency bands. The stimuli of rainbow colors are depicted under the panels. The values were depicted as means ± SEMs. (**D**,**E**,**F**) display the means of WPLIs between 0 and 500 ms without differentiating the rainbow-color-stimuli. *Denotes the *p* value < 0.05. (one-way ANOVA, then Bonferroni post hoc comparison; stimulation trials as the unit to be analysis).

We analyzed the phase synchronizations between ROT and NCL (ROT-NCL), ENTO and NCL (ENTO-NCL), and VW and NCL (VW-NCL) by the weighted phase lag index (WPLI) to determine whether the higher-level cognitive-related brain region, the NCL, involves in processing color stimuli. The WPLI is an index which is insensitive to volume conduction^44^ and suitable to analyze the levels of synchronization within a small size region of brain. We calculated WPLI within 500 ms after different color stimuli (Fig. 4A–C) and we also determined WPLI within 500 ms by all of rainbow color stimuli (Fig. 4D–F). The phase synchronization of the ROT-NCL, ENTO-NCL, and VW-NCL were not significantly higher compared with other colors when stimulated with blue (Fig. 4A–C; analyzed by one-way repeated measures ANOVA compared then Bonferroni-adjusted significance tests for pairwise comparisons. See Table S5 for detailed statistic values). However, the ENTO-NCL pathway showed significant higher WPLI in all colors when compared with those of VW-NCL, indicating more prominent increases between NCL and relay nuclei in the tectofugal pathway than that between NCL and VW of the thalamofugal pathway (Fig. 4D–F; one-way ANOVA, Bonferroni-adjusted significance tests for pairwise comparisons. See Table S6A for detailed statistic values). One-way repeated measures ANOVA were also used and demonstrated higher ENTO-NCL synchronization than VW-NCL in middle and high frequency (Fig. S5C, Table S6B for detailed statistic values). Again, blue was extracted and further analyzed (Fig. S5D, Table S6C for detailed statistic values; One-way repeated measures ANOVA). The results demonstrated significant stronger phase synchronization in ENTO-NCL than that of VW-NCL in the high frequency. These results imply that the color signal transmission was mediated by the tectofugal pathway to NCL. To further confirm the transmission pathway and connectivity, we injected the non-trans-synaptic retrograded tracer, fluorogold, into NCL to confirm the anatomical afferent projections to NCL (Fig. S6A–C). The brain histology showed that the bilateral ENTOs exhibit prominent fluorescent signals after injecting fluorogold bilaterally to NCL. Projections from the striatum were also noticed. In contrast, there was a mild retrograded signal in the caudal part of VWs (Fig. S6B). To determine the hemispheric integration of these pathways, we injected fluorogold into the left NCL and incubated for additional two weeks. We found that most of the signals were noticed in the mesopallium, and there was few fluorogold signal in the left cranial part of VW (Fig. S6C). This retrograde staining result implies that the afferent projection to NCL is predominant from ENTO, but not from VW.

### Color information is processed in the direction from tectofugal pathway to NCL

**Figure 5:**
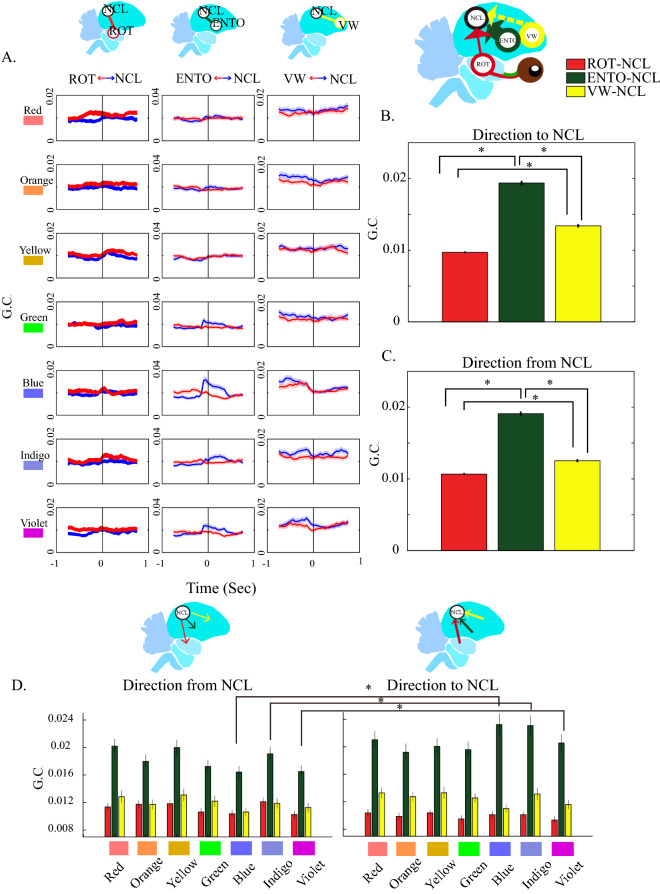
Granger causality between the NCL and other 3 brain regions after stimulated by rainbow colors with black color as baseline between each color. (**A**) The dynamics of Granger causality between the NCL and other 3 regions. The data in the y-axis are the value of Granger causality before and after stimulating with rainbow colors. The means ± SEMs are displayed by solid lines and shades, where blue lines are Granger causality direction from each 3 regions to the NCL and red lines are the opposite direction. Please note that it must sample a period of time for calculating the Granger causality. We took every 0.5 s with 0.05 s moving steps to measure the dynamics (see the Method section); therefore, the time resolution was not as precise as the LFP traces and resulted in the peak G.C.s before 0 s. And also, the scale for ENTO-NCL is different for others (**B**,**C**) show the mean G.C. between 0 and 500 ms without differentiating the rainbow-color-stimuli. The values were depicted as means ± SEMs. *Denotes the *p* value < 0.05. (one-way ANOVA, then Bonferroni post hoc comparison; stimulation trials as the unit to be analysis). (**D**) represents the mean of G.C. between 0 and 500 ms after the stimulation of rainbow colors. The values were depicted as means ± SEMs. *Denotes the *p* value < 0.05. (paired t test).

We further assessed the functional connectivity between the two pathways and NCL to reveal the directional interactions when the subjects were stimulated by different colors (Fig. 5A). The overall Granger causality for both directions are significantly higher in the ENTO ⇔ NCL (Fig. 5B,C, Table S7A for detailed statistic values and Bonferroni *post-hoc* tests of one-way ANOVA). One-way repeated ANOVA demonstrated the significant strongest Granger causality in the ENTO ⇔ NCL as well if we pooled all the results of all rainbow colors (Fig. S7A, Table S7B for detailed statistic values). This phenomenon is still significant if we only analyzed blue stimuli (Fig. S7B, Table S7C for detailed statistic values). The increase of Granger causality was observed in a direction from ENTO to NCL during blue stimuli (Fig. 5D, two-tailed paired *t*-test, see Table S8B for detailed degree of freedom, t values and *p* values). Similar finding could also be found after indigo (Fig. 5D, Table S8B) and violet stimuli (Fig. 5D, Table S8B), but not after other color stimuli. These results suggest that the LFPs recorded from ENTO are leading the LFPs in NCL, because the direction from ENTO to NCL demonstrated the highest Granger causality and implied the color information is processed from the tectofugal pathway to NCL. Finally, it is also interesting to determine whether ROT transfers information to ENTO, because ROT is a relay nucleus of tectofugal pathway. Our data demonstrated that the direction from ROT to ENTO had a higher Granger causality than that of the direction from ENTO to ROT after the stimulation of rainbow colors (Fig. S7C, Table S7D for detailed statistic values) or blue color (Fig. S7D, Table S7E for detailed statistic values).

## Discussion

ENTO and VW are respectively the telencephalic areas of tectofugal pathway and thalamofugal pathway. However, it is still unclear whether higher order avian brain areas, such as NCL, communicate with them or not. We hypothesized that the telencephalons of visual pathways communicate with higher order brain areas in zebra finches and the ENTO has stronger communication with the NCL than VW, when stimulated by colors. We initially were expecting prominent responses in ENTO when zebra finches see colors. The present study demonstrated that the ENTO generates stronger power than those of ROT and VW when the right eye was stimulated by colors. In addition, the NCL also represented strong power. When we further analyzed the functional connectivity between ENTO and NCL, we found that color stimuli enhanced synchronization between ENTO and NCL in the direction from ENTO to NCL. Interestingly, the synchronization between VW and NCL were negatively synchronized, suggesting these two areas communicated poorly with each other^37^. Moreover, blue is the color that evoked the strongest power in the direction from ENTO to NCL; the Granger causality also demonstrated the LFPs acquired from ROT leaded the consequent LFPs of ENTO. However, the different colors did not result in different phase synchronization between NCL and other three regions. We were also interested in learning what colors zebra finches are sensitive to. Indeed, we hypothesized that their visual pathway is sensitive to red, but we cannot ignore the fact that blue is important for finding sky or water and green may be important for searching plants. Surprisingly our results partially support that blue is relatively important to zebra finches. These results also raise some interesting questions about the physiological or ecological advantages of “blue” and also the physiological functions of the tectofugal pathway, the thalamofugal pathway, and NCL for zebra finches. The LFP experiments are mainly done in rodent models; therefore, we firstly applied findings from rodents to explain our current results. Then, we discussed some potential physiological meanings for zebra finches.

### The LFP spectrograms

We employed the results of LFPs to determine our hypothesis. The LFP is a summation of various potentials such as membrane potential and action potentials. The synaptic potential is often the main source of LFP^38^. The LFP contains multi-dimensional information including frequency, amplitude, phase, and time. Therefore, the present study analyzed several aspects of the LFPs. In the spectrograms, we noticed that blue evoked a strongest power within 0–500 ms after the stimuli, especially in ENTO and NCL. Strong LFP power (or amplitude) usually suggests that the target areas are in a very active status^38^. In addition, the color stimuli evoked approximately three frequency bands. The LFP frequency is an unique way for communicating between brain regions and each frequency band may encode different information and has its own physiological functions^39^. We analyzed the frequency bands to explore the potential communication of color information between ENTO and NCL. Our data demonstrated that the middle and high frequency bands revealed the power differences between ENTO and VW. In general, low frequency oscillations are propagated farther, given that the cell membrane is a low pass filter^37^. In the other hand, high frequency oscillations provide a more temporally organized transmission than low frequency oscillations^40,41^. We cannot conclude that the color information is not transmitted between VW and NCL, since we still acquired significant potentials in VW. However, the evidence from phase synchronization and Granger causality support that the ENTO-NCL is more sensitive to colors.

### Phase synchronization and directional connectivity

The phase of LFP also encodes information for propagating between brain regions^37^. Synchronizations help the communication between different brain regions^45^. Although we found no color preference of phase synchronization, the ENTO-NCL still revealed strong positive synchronization, whereas VW-NCL showed weak or negative synchronization. Even though the blue evoked the highest activities in the ENTO and NCL, the WPLI cannot reflect the difference of amplitudes because WPLI only measures the phases of LFPs. We postulated that the phase synchronization encoded information of stimuli, but not colors. In the other word, the communications between ENTO and NCL increased, which paved a way for color information but the color itself was encoded by amplitudes. The weak communication between VW-NCL implys that the tectofugal pathway is the main path in response to color stimulation. The directional connectivity also suggests a strong information transmits from ENTO to NCL when zebra finch received blue stimulation.

### Sensitivity to blue stimulation

We demonstrated that the male zebra finch is sensitive to blue lights. The retina studies have shown that several species of birds are sensitive to both yellow and UV lights, but some species show their maximal sensitivity at the green spectrum^46^. Therefore, the spectral sensitivity in birds may vary among species. Bennett et. al. reported that UV vision dominantly contributes to the mate-selection in zebra finches^47^. Our data could not rule out the possibility of sensitivity for UV light in the zebra finch, but we believe our results shed some light on zebra finches’ color preference. Additionally, a recent study also demonstrates the zebra finches’ retina is sensitive to blue as well^48^.

Our results also showed that the color-evoked potentials were significantly detected when we used black as baseline, rather than white color, between different colors. It is possible that white baseline, which contains all spectra of the visible lights, may saturate the visual response and let the visual pathways do not respond to the subsequent colors. There is also a behavior report indicates that the contrast of the background affects color discrimination in zebra finches^49^. Although the background used in the present study are not parallel to the back or white baseline, it is likely that the responses to colors are modulated by recent or adjacent colors. To minimize the confounding factor, we stimulated the subjects with colors in random orders. The intensities of white or other colors may also affect the brain activities. A study regarding the spectral sensitivity in avian retina reports that increasing intensity of certain color shifts the maximum electroretinal potential toward the shorter wavelengths^46^. Therefore, we manually adjusted the intensities of different colors to be the same and acquired their evoked potentials. The limitation of this study is the light source, since we did not use a light with narrow wavelength band such as laser. We simply used a laptop, which generates colors based on a human’s vision system (i.e. trichromatic colors). Even though this study did not stimulate the visual pathway with a precisely narrow wavelength band and no invisible light (for humans) was generated by the laptop, the results still shed light on their brain activities when stimulated by trichromatic colors. We expected more complicated responses will be observed when stimulated by mixing the UV light with trichromatic colors^47^.

In addition, the color perception in zebra finches is a complicated issue. Our report raises several new questions needed to be discuss. For instances, what the limitation is for using a monitor which generates RGB colors to stimulate the animals with tetrachromatic vision. Do colors influence the zebra finches’ perceptive brightness and cause the strongest evoked LFPs from blue? Although the functions of the avian brain are species-specific, we reviewed some studies from other bird species and tried to reveal the potential answers of whether the violet cones (or ultraviolet cones in finches^48^) interact with the S-, M-, and L-cones in the tetrachromatic vision of bird. For humans, the purple from the monitor stimulates both red- and blue-sensitive cone cells and humans interpret it as purple^50^. The purple from the laptop monitor is a mixing of red and blue, not a pure short wavelength light. Therefore, we should roughly interpret the rainbow colors as: red, red + green (which generate orange and yellow), green, blue, blue + red (which generate indigo and violet) (Table S2 for the detail combination of RGB). Recently, a study from hummingbirds constructs an avian tetrahedral color space^51^ and proves their ability to discriminate UV. Our study just implied that the stimulation on the zebra finch S-cones evoked a strong response, but still cannot rule out the possibility that UV can generate strongest visual responses in their brain. On the other hand, despite we adjusted the rainbow colors to have the same radiation power, it is still unclear how blue color generates strongest responses in the brain. In human, we feel yellow is brighter than red, green, blue, even they are displayed by the same radiation power. We proposed that the mechanism of feeling different brightness among colors in human is not the cause of strong blue response in the zebra finch. The sensitivity spectrum of human’s green- and red-sensitive cone cells is highly overlapped^52^. Therefore, yellow is able to stimulate more cones than other colors. But the sensitivities of zebra finches’ cone cells are evenly distributed across spectrum^48^. Thus, we think the strong blue response is not a phenomenon of cross-reaction between different types of cone cells. Indeed, study from the oil droplets of zebra finches implies that zebra finches have higher cone spectral sensitivities for blue and UV than red and green^48^. Our data obtained from the brain activities further support this result from the retina’s study.

Although present study did not explain why the brain of zebra finch is more active to blue, their nature habitat may hint the potential reasons. Zebra finch is a diurnal animal and lives in relative arid areas^53^. We hypothesized that water and sky are key factors for zebra finch surviving, so they need to be spotted as fast as possible. During the experiment we also observed that zebra finches became quite and standstill if we turn off the room light. We think, as the room light was off, zebra finches were searching for sky for flying toward. Besides, researchers discover that zebra finches have blue and UV light-dependent magnetic compass, suggesting that blue light is critical for their navigation^16^.

### Tectofugal pathway, thalamofugal pathway and NCL

Our findings indicated that the color-evoked potentials were stronger in the tectofugal pathway than the thalamofugal pathway. Moreover, the synchronization between the relayed nuclei of the tectofugal pathway and NCL was also stronger. These results are similar to some studies using pigeons as subjects, in which lesion of ROTs impairs the color discrimination^9^ and some color sensitive units are also found in ROT^54^. In addition to ROT, about 30% of tectal units are able to respond to certain wavelengths^17^. Although we discovered that ENTO (a downstream of ROT) represents stronger activities than those of ROT, we cannot exclude the roles of ROT for processing color information. In our result, we demonstrated that NCL responses were highly correlated with ENTO but the correlation is relatively low between the NCL and ROT. We think ROT did not reveal as strong response as ENTO because ENTO needs more intensive communication with NCL. This hypothesis is supported by the synchronization and directional connectivity results of NCL-ENTO. With regard to the thalamofugal pathway, color-sensitive units have been discovered in the ventral lateral geniculate nucleus, which consists of inputs from both retinas and VW^55^. Bredenkotter and Bischof used 1-ms flash to evoke and record the field potentials of VW and ENTO, and found the amplitudes recorded from the contralateral hemispheres are similar^56^. A lesion study demonstrates that the VW in zebra finch involves in spatial information processing and ENTO analyzes the pattern of objects^57^. The zebra finches’ VW even perceive vision mediated by earth magnetic field orientation^58^. It is still unclear whether the VW modulates other cognitive functions related to vision, but researchers demonstrate the important role of VW in imprinting for chicken^59^. These pieces of evidence may support our hypothesis that the tectofugal pathway is much critical than the thalamofugal pathway in regard to the color information processing, since colors are also important cues for discriminating objects. It would be also of interest to simultaneously record from the ventral lateral geniculate nucleus. However, because of the limitations of channel and skull space, we selected only to record the relay nucleus (ROT) of tectofugal pathway.

## Conclusions

Our result suggests that the communication between nidopallium caudolateral and tectofugal pathways is crucial for color discrimination. Moreover, ENTO and NCL are more active when the eyes are stimulated by blue and the visual information was transmitted from the direction of ENTO to NCL.

## Materials and methods

### Animals

In the experiments, the male zebra finches (n = 9, 5 to 8-month-old) were acquired from the commercial bird breeders (San-Xing Bird Store, Taipei, Taiwan). The birds were housed in home cages individually. The temperature of the environment temperature was controlled at 23 ± 1 °C, and the light–dark cycle was maintained under nature light (AM 7:00 light, PM 7:00 dark; summer). Food and water were available ad libitum. All procedures performed in this study were approved by the National Taiwan University Animal Care and Use Committee, approval ID: NTU-106-EL-026. All methods described in this paper were performed in accordance with the guidelines and regulations of National Taiwan University Animal Care and Use Committee.

### Surgery and electrophysiological data collection

After at least a 7-day accommodation in their home cages, the zebra finches were randomly selected for the recordings of evoke potentials. These finches stayed in an induction box, which provided pure oxygen for 10 min to raise their blood oxygen levels. Subsequently, they were intraperitoneally administrated butophanol (2 µg/g) and midazolam (2 µg/g) for analgesia and muscle relaxing. Anesthesia was induced by 2% isoflurane mixed with oxygen. Once they lost their reflex of deep pain, the subjects were fixed on the stereotaxic instrument and maintained anesthesia with 1.5% isoflurane^60^. Four tetrodes, consisting of two twisted 0.05 mm stainless steel wires (California Fine Wire, Grover Beach, CA) in each one, were implanted into the NCL (AP, 1.0 mm; ML, − 4.5 mm; DV, 4.0 mm relative to y point), the ENTO (AP, 3.0 mm; ML, − 3.6 mm; DV, 3.3 mm relative to y point), and the ROT (AP, 2.6 mm; ML, − 2.0 mm; DV, 5.0 mm relative to y point), and the VW (AP, 5.5 mm; ML, − 2.0 mm; DV, 1.5 mm relative to y point) in the left hemisphere. The coordinates were selected based on *A stereotaxic atlas of the brain of the zebra finch* by Nixdorf-Bergweiler and Bischof^61^. The tetrodes were connected to an interface board which linked to a head stage and tether to the preamplifier of OmniPlex A system (omniplex version 1.2.0, https://plexon.com/products/omniplex-software, Plexon, Dallas, TX, USA). One grounding screw was anchored on the rostral part of right frontal skull. During the recording, the grounding electrode was used as a reference and the LFP signals were amplified, digitalized, and recorded by a 16 channels OmniPlex A system. The raw LFP signals were bandpass filtered between 0 and 500, amplification time was set at 2500, and the sampling rate was 40 kHz. These digitalized data then down sampled to 2000 Hz for storage. Some reports demonstrate that zebra finches use their right eye to choose their mates which have colorful beaks and feathers^28^. To test if the left visual pathways of zebra finches are sensitive to certain colors, we flashed 15 colors or 7 rainbow colors to the right eyes of birds. The eyelids of the right eyes were opened and fixed with #6-0 surgical sutures. The color flashing time stamps were also integrated and stored in OmniPlex A system by commercial recording software OmniPlex (version 1.2.0, Plexon). For detailed color flashing method, see the section of color flashing. All of the post-recording data were analyzed by custom written code in MATLAB R2016b (MathWorks, Natick, MA, USA).

### Color flashing

Fifteen colors were generated using MATLAB code: jet(15), which makes RGB codes from blue to green to red. (The RGB color codes are: (0, 0, 0.75), (0, 0, 1), (0, 0.25, 1), (0, 0.5, 1), (0, 0.75, 1), (0, 1, 1), (0.25, 1, 0.75), (0.5, 1, 0.5), (0.75, 1, 0.25), (1, 1, 0), (1, 0.75, 0), (1, 0.5, 0), (1, 0.25, 0), (1, 0, 0), and (0.75, 0, 0)). We further created 7 rainbow colors with the same radiation power (Table S1), because the 15 colors did not generate the same radiation power. The RGB color codes of the rainbow colors are: red: (1, 0.47, 0.47), orange: (1, 0.58, 0), yellow: (0.86, 0.68, 0), green: (0, 1, 0), blue: (0.4, 0.4, 1), indigo: (0.51, 0.54, 0.87), and violet: (0.85, 0, 0.85). The intensity of radiation powers (Table S1) were confirmed by light meter (model: MR-16, RAINBOW-LIGHT, Taiwan). For the comparison between these colors and black/white colors, four kinds of color sequences, which are black order (BO), black random (BR), white order (WO), and white random (WR), were used in the experiment. The color sequences in the BO were used above mentioned sequence of color codes (either 7 or 15 colors) and inserted black color between colors. The pattern of BR is to rule out the confounding factor caused by the order of colors. We randomly picked 15 (or 7) colors without regular sequence and inserted black color between colors. The color sequences of WO and WR are similar to BO and BR, except that we inserted white color between colors. These colors were flashed on a laptop (model: SVP132A1CP, SONY) placed in front of the subjects’ right eyes (the monitor was approximately 30 cm away from the subject). Each color, including black or white colors, emerged for 2 s and then switched to another color in a sequence (order or random) as described earlier. Each subject received a total of 80-time stimuli of color sequences (20 trials in each BO, BR, WO, and WR). The pilot tests had indicated that zebra finches sobered up when isoflurane was below 1%; thus, we maintained the concentration of isoflurane between 1 and 1.5% during the whole experiment. At the end of the experiment, we euthanized the subject with intraperitoneal administration of Zoletil (Tiletamine:Zolazepam = 1:1 Virbac, Carros, France). Their brains were collected and soaked in 10% formalin to confirm the location of implanted electrodes.

### LFP analysis

We extracted the time stamps of every color-changing point and extended the time ± 1 s. The local field potentials in the ± 1-s periods were used for further analysis as described below.

#### Evoked potentials (LFPs) and power estimation

The LFPs were Z-scored and averaged across the same color for measuring the evoked potentials, and the negative Z-scored power means it is below the average power. The power spectrograms were analyzed with the multitaper method from the open-source MATLAB toolbox Chronux^62^. Because the spectrograms need a period of samples and step a short time to create a dynamic of spectrum as function of time. We used 0.5-s windows with 0.05-s overlapping steps, set the time-bandwidth product at 3, and set the number of tapers at 5. We also extracted and averaged the values of stimuli between 0 and 500 ms for testing the statistic differences among stimuli or brain regions.

#### Phase synchronization across regions

The levels of synchronization between the ROT-NCL, ENTO-NCL, and VW-NCL were evaluated with a debiased estimator of the squared weighted phase lag index (WPLI)^44^. The codes can be download from the open source tool box, Fieldtrip https://www.fieldtriptoolbox.org/download.php^63^. The WPLI analyzes an imaginary component of the spectrum across two LFPs, because it not only relates to the phase synchronization between two LFPs but is also insensitive to noise or contamination from volume conduction. Compared with classical coherence, this estimator analyzes the phase synchronization across brain regions and minimizes the effect of volume conduction contamination and sample size bias^44^. The WPLI normalized the two LFP with perfect synchronization to 1 and completed out of phase to -1. In order to access the WPLI in a similar manner with multitaper power estimation, we also broke the ± 1-s periods into 0.5-s windows with 0.05-s step and estimated the WPLIs. We extracted and averaged the values of each stimulation during 0–500 ms for testing the statistic differences among stimuli or brain regions.

#### Granger causality

The function connectivity between the ROT⇔NCL, ENTO⇔NCL, and VW⇔NCL were accessed by using Granger causality. We adapted the open-source MATLAB toolbox developed by Barnett and Seth^64^. It is also due to the input samples must contain a period of time to generated a Granger causality dynamic over time, which is similar to the method described in the power spectrogram and the WPLI, we calculated the Granger causality for the time domain every 0.5 s with 0.05-s steps for measuring the dynamics of Granger causality. We extracted and averaged the values of each stimulation during 0–500 ms for testing the statistic differences among stimuli or brain regions.

### Histology and retrograde tracing of the afferents to the NCL

For tracing the afferent projections to NCL, a retrograde tracer, 4% fluorogold (Sigma Chemical), was microinjected into NCL in two birds. One subject was bilaterally (the NCL; AP, 1.0 mm; ML, ± 4.5 mm; DV, 4.0 mm relative to y point) injected with fluorogold using microinjection syringe pump at a speed of 1 µl/10 min. We administered 0.33 µl of fluorogold at each site and waited for 1 min, then moved the tip of the needle up for 100 µm and administered another 0.33 µl again and waited another 1 min, and repeated the procedure once again. Two weeks after injections, this bird was euthanized by isoflurane. The brain was removed and dissected into a 30-µm coronal section by a cryostat microtome. Some brain slices were stained with DAPI. We used an ultraviolet filter in the inverted microscope (IX83; Olympus, Tokyo, Japan) to detect the fluorescent reaction of fluorogold. Since the fluorescent reaction in VW was not high in this bird (Fig. S6B), we double confirmed this result with anther finch, which was microinjected with 4% fluorogold to the left NCL and waited for 4 weeks to let fluorogold travel a longer distance. The brain slices for confirming the locations of the implanted electrodes were also dissected into a 30-µm coronal section by the cryostat microtome. The pictures in Fig. S6E–H were taken under stereo microscope without staining. The coordinates were adopted from *A stereotaxic atlas of the brain of the zebra finch*^61^.

## Results and statistics

All results in the figures are depicted as the means ± SEMs. The results of statistical analyses were done by SPSS (Version: 10.0.7, IBM, New York, USA). The stimulation trials were used as unit to be analyzed. We initially used one-way ANVOA to compared the differences between brain areas. In addition, we excluded the miss values of VW in one bird and retested differences between brain areas with one-way repeated measures ANOVA. Bonferroni post hoc comparison was used if the ANOVA test indicates a significant difference. For measuring the difference between the two causality directions, we used two-tailed paired t-tests.

## Supplementary information

Supplementary Legends.

Supplementary Figure 1.

Supplementary Figure 2.

Supplementary Figure 3.

Supplementary Figure 4.

Supplementary Figure 5.

Supplementary Figure 6.

Supplementary Figure 7.

Supplementary Tables.
