# Supplementary material for: Connectivity between nidopallium caudolateral and visual pathways in color perception of zebra finches
Source: Sci Rep. 2020 Nov 9;10:19382. doi: 10.1038/s41598-020-76542-z (PMC7653952; doi:10.1038/s41598-020-76542-z)
Supplement: Supplementary file 1 — Supplementary Legends. [file 41598_2020_76542_MOESM1_ESM.docx]

**Connectivity between nidopallium caudolateral and visual pathways in color perception of zebra finches**

Yi-Tse Hsiao^1^, Ta-Ching Chen^2,3§^, Pin-Huan Yu^4^, Ding-Siang Huang^2^, Fung-Rong Hu^2^, Cheng-Ming Chuong^5^, Fang-Chia Chang^1,6,7,8*^

^1^ Department of Veterinary Medicine, School of Veterinary Medicine, National Taiwan University, Taipei, Taiwan

^2^ Department of Ophthalmology, College of Medicine, National Taiwan University, Taipei, Taiwan

^3^ Research Center for Developmental Biology and Regenerative Medicine, National Taiwan University

^4^ Institute of Veterinary Clinical Science, School of Veterinary Medicine, National Taiwan University, Taipei,

Taiwan

^5^ Department of Pathology, University of Southern California, Los Angeles, California, USA

^6^ Graduate Institute of Brain & Mind Sciences, College of Medicine, National Taiwan University, Taipei, Taiwan

^7^ Graduate Institute of Acupuncture Science, College of Chinese Medicine, China Medical University, Taichung, Taiwan

^8^ Department of Medicine, College of Medicine, China Medical University, Taichung, Taiwan

**Supplementary figure legends**

Figure S1. **The averaged LFP traces in the brain regions after stimulated by 15 colors with black color as baseline between each color.** The LFP amplitudes of y-axis were Z-scored and depicted as the means ± SEMs. The zero at the x-axis is the stimulation time point.

Figure S2. **The averaged spectrograms in the brain regions after stimulated by 15 colors with black color as baseline between each color.**  Frequencies (Hz) have shown on the y-axis and stimulation time point is marked as zero at the x-axis. The magnitude of power is color-coded and the power scales are plotted under each column.

Figure S3. **The averaged spectrograms in the brain regions after stimulated by 15 colors with white color as baseline between each color.**  Frequencies (Hz) have shown on the y-axis and stimulation time point is marked as zero at the x-axis. The magnitude of power is color-coded and the power scales are plotted under each column.

Figure S4. **The averaged LFP traces in the brain regions after stimulated by rainbow colors with black color as baseline between each color.** The LFP amplitudes of y-axis were Z-scored and depicted as the means ± SEMs. The zero at the x-axis is the stimulation time point.

Figure S5. **The averaged spectrograms and WPLI in the brain regions after stimulated by rainbow colors or blue with black color as baseline between each color.** (A) Averaged powers stimulated by rainbow colors within the time period (0 to 500 ms) of low (A1), middle (A2), and high frequency bands (A3) and compared between 4 brain areas. (B) Averaged powers stimulated by blue within the time period (0 to 500 ms) of low (B1), middle (B2), and high frequency bands (B3) and compared between 4 brain areas. (C) Averaged WPLI stimulated by rainbow colors within the time period (0 to 500 ms) of low (C1), middle (C2), and high frequency bands (C3) and compared between 4 brain areas. (D) Averaged WPLI stimulated by blue within the time period (0 to 500 ms) of low (D1), middle (D2), and high frequency bands (D3) and compared between 4 brain areas. The values were depicted as means ± SEMs. * denotes the *p* value < 0.05. (one-way repeated measures ANOVA, then Bonferroni post hoc comparison; stimulation trials as the unit to be analyzed).

Figure S6. **The brain slices after bilateral or unilateral infusing fluorogold into the NCL and examples of electrode implantation targets**. (A) and (B) are the slices obtained from bilateral administration of fluorogold. (C) represents slices obtained after left NCL injection of fluorogold. Scale bars indicate 1 mm. The blue mark is the NCL, red marks are the hyperpallium apicale of VW, yellow mark is the hyperpallium densocellulare of VW, black marks are the ENTO, and the green marks are the ROT on the right side of drawing atlas diagrams. (D) represents the slice sections in A, B and C panels. The signals in the cranial and caudal VW were weak. (E), (F), (G), and (H) are examples of recording sites. The red arrows indicate the deepest lesions of tetrodes which are the recording targets. The blue arrow points out a midway of tetrode which is ending at the ROT. (I) demonstrates the slice sections in E, F, G and H panels. Abbreviations: A: Arcopallium, BSTL: Lateral part of the bed nucleus of the stria terminalis, Cb: Cerebellum, CT: Commissura tectalis, DA: Tractus dorso-arcopallialis, DBC: Decussatio brachiorum conjunctivorum, FLM: Fasciculus longitudinalis medialis, FPL: Fasciculus prosencephali lateralis (lateral forebrain bundle, FRL: Formatio reticularis lateralis mesencephalic, GLV: Nucleus geniculatus lateralis, pars ventralis, GP: Globus pallidus, HA: Hyperpallium apicale, HP: Hippocampal formation, HVC: formal name, located in nidopallium, ICo: Nucleus intercollicularis, ICT: Nucleus intercalatus thalami, LaM: Lamina mesopallialis, LFS: Lamina frontalis superior, LM: Nucleus lentiformis mesencephalic, LPS: Lamina pallio-subpallialis, M: Mesopallium, MLd: Nucleus mesencephalicus lateralis, pars dorsalis, N: Nidopallium, NIV: Nervus trochlearis, OM: Tractus occipitomesencephalicus, OMd: Nucleus nervi oculomotorii, pars dorsalis, OMv: Nucleus nervi oculomotorii, pars ventralis, PL: Nucleus pontis lateralis, PV: Nucleus posteroventralis thalami, Rt: Nucleus rotundus, StL: Striatum laterale, TeO: Tectum opticum, TFM: Tractus thalamo-frontalis et frontalis-thalamicus medialis, TrSM: Tractus septopalliomesencephalicus, TrO: Tractus opticus, TT: Tractus tectothalamicus, V: Ventricle. Adopted from *A stereotaxic atlas of the brain of the zebra finch* by Nixdorf-Bergweiler and Bischof ^64^.

Figure S7. **The averaged Granger causality** **between the NCL and other 3 brain regions after stimulated by rainbow colors or blue with black color as baseline between each color. (**A) The means of G.C. between 0 and 500 ms without differentiating the rainbow-color-stimuli. (A1) Direction from NCL to ROT, ENTO, or VW. (A2) Direction from ROT, ENTO, or VW to NCL. (B) The means of G.C. between 0 and 500 ms after being stimulated by blue. (B1) Direction from NCL to ROT, ENTO, or VW. (B2) Direction from ROT, ENTO, or VW to NCL. (C) are averaged G.C. between the ENTO and ROT after being stimulated by rainbow colors. (D) are averaged G.C between the ENTO and ROT after being stimulated by blue. The values were depicted as means ± SEMs. * denotes the *p* value < 0.05. (one-way repeated measures ANOVA, then Bonferroni post hoc comparison; stimulation trials as the unit to be analyzed).

Table S1. The intensities and RGB codes of different colors, black and white colors. We adjusted the rainbow colors to a similar VIS radiation power.

Table S2. **Detailed statistical results of z-scored power.** Compared between 15 colors. Done by one-way repeated measures ANOVA, Bonferroni-adjusted significance tests for pairwise comparisons. *p* < 0.05 are labeled by red.

Table S3. **Detailed statistical results of z-scored power when compared between four brain areas.** (A). After pooling the power of 15 colors. Done by one-way ANOVA. (B). After pooling the power of Rainbow colors. Done by one-way ANOVA. (C) Done by one-way repeated measures ANOVA and some trails with missing values were excluded. (D). Analysis of the data from blue stimulation. Done by one-way repeated measures ANOVA and some trails with missing values were excluded. Bonferroni-adjusted significance tests for pairwise comparisons. *p* < 0.05 are labeled by red.

Table S4. **Detailed statistical results of z-scored power.** Compared between rainbow colors. Done by one-way repeated measures ANOVA, Bonferroni-adjusted significance tests for pairwise comparisons. *p* < 0.05 are labeled by red.

Table S5. **Detailed statistical results of WPLI.** Compared between rainbow colors. Done by one-way repeated measures ANOVA, Bonferroni-adjusted significance tests for pairwise comparisons. *p* < 0.05 are labeled by red.

Table S6. **Detailed statistical results of WPLI when compared between ROT-NCL, ENTO-NCL, and VW-NCL.** (A). After pooling the power of rainbow colors. Done by one-way ANOVA. (B) Done by one-way repeated measures ANOVA and some trails with missing values were excluded. (C). Analysis of the data from blue stimulation. Done by one-way repeated measures ANOVA and some trails with missing values were excluded. Bonferroni-adjusted significance tests for pairwise comparisons. *p* < 0.05 are labeled by red.

Table S7. **Detailed statistical results of Granger causality (G.C.) when compared between ROT⬄NCL, ENTO⬄NCL, VW⬄NCL.** (A). After pooling the power of rainbow colors. Done by one-way ANOVA. (B) Done by one-way repeated measures ANOVA and some trails with missing values were excluded. (C). Analysis of the data from blue stimulation. Done by one-way repeated measures ANOVA and some trails with missing values were excluded. (D). G.C. values of ROT to ENTO and ENTO to ROT after being stimulated by rainbow colors (E). G.C. values of ROT to ENTO and ENTO to ROT after being stimulated by blue. Bonferroni-adjusted significance tests for pairwise comparisons. *p* < 0.05 are labeled by red. Bonferroni-adjusted significance tests for pairwise comparisons. *p* < 0.05 are labeled by red.

Table S8. Granger causality directional analysis after stimulating by rainbow colors. (A) ROT to NCL vs. NCL to ROT. (B) ENTO to NCL vs. NCL to ENTO. (C) VW to NCL vs. NCL to VW. Two tailed paired *t*-test. P < 0.05 are labeled by red.
