# Supplementary figures and images for: Connectivity between nidopallium caudolateral and visual pathways in color perception of zebra finches
Source: Sci Rep. 2020 Nov 9;10:19382. doi: 10.1038/s41598-020-76542-z (PMC7653952; doi:10.1038/s41598-020-76542-z)

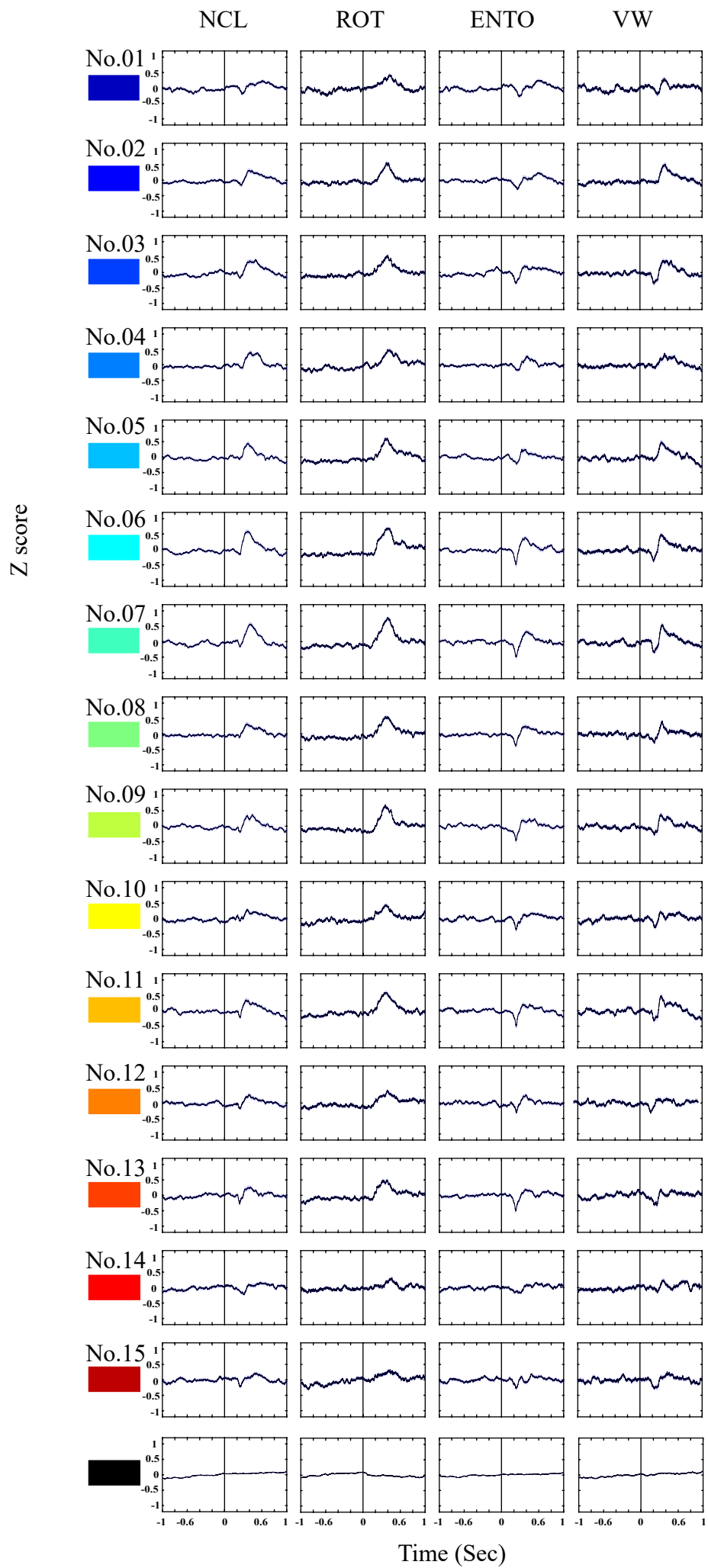

Supplement: Supplementary file 2 — Supplementary Figure 1. [file 41598_2020_76542_MOESM2_ESM.pdf]

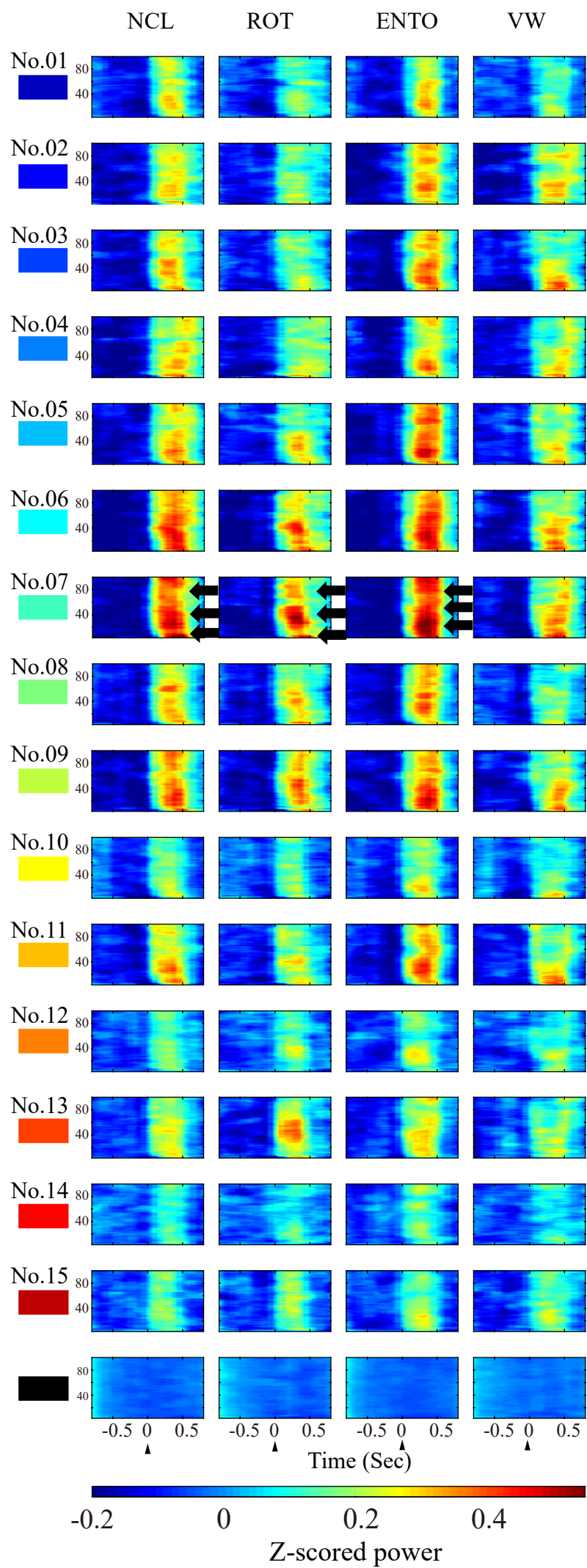

Supplement: Supplementary file 3 — Supplementary Figure 2. [file 41598_2020_76542_MOESM3_ESM.pdf]

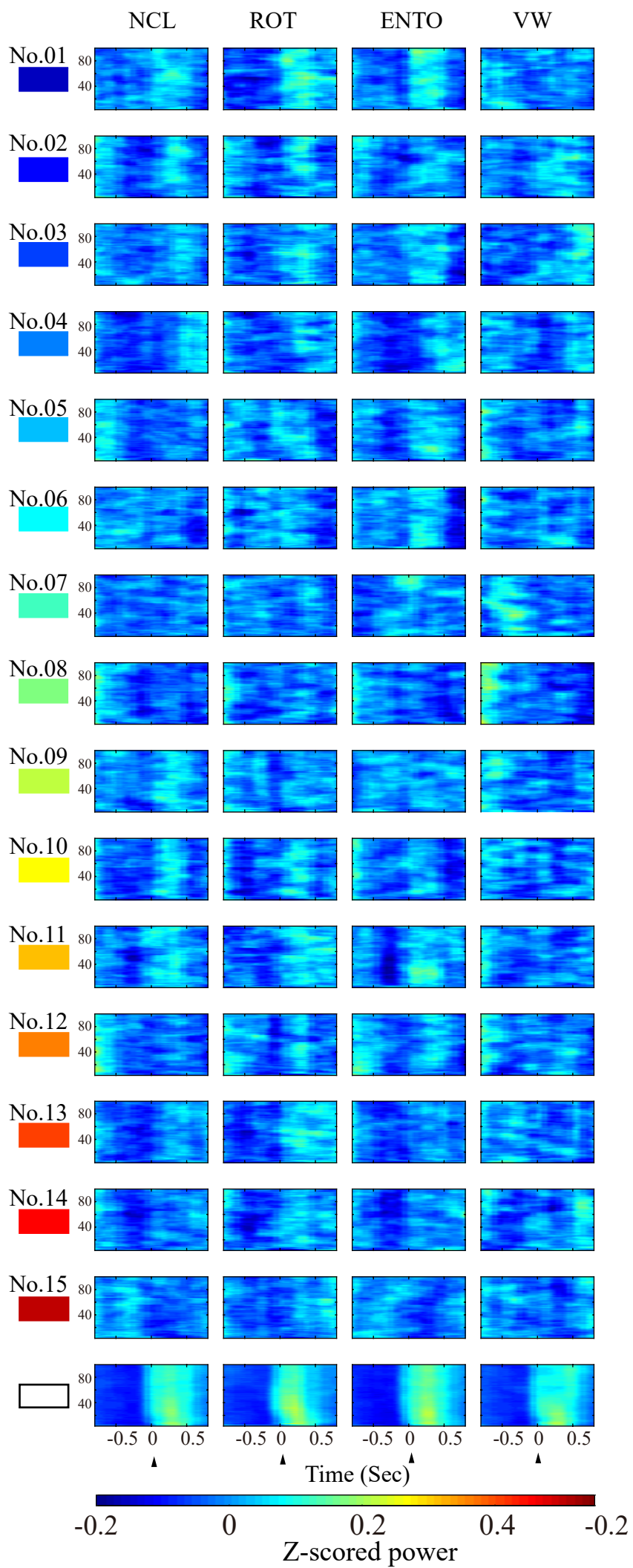

Supplement: Supplementary file 4 — Supplementary Figure 3. [file 41598_2020_76542_MOESM4_ESM.pdf]

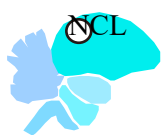

NCL

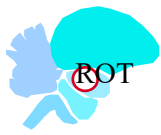

ROT

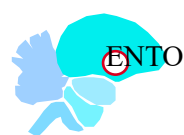

ENTO

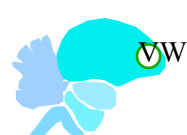

VW

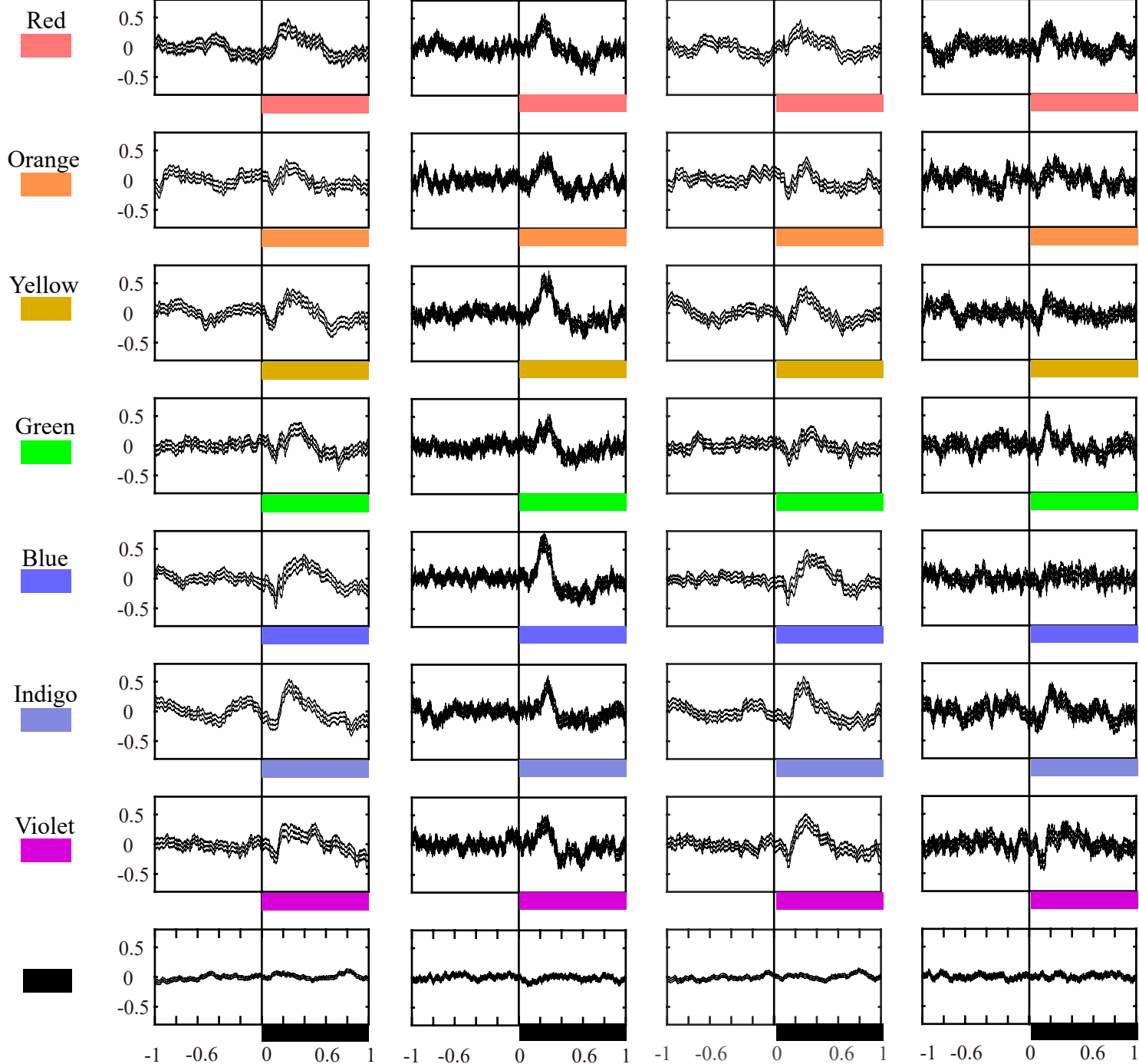

Supplement: Supplementary file 5 — Supplementary Figure 4. [file 41598_2020_76542_MOESM5_ESM.pdf]

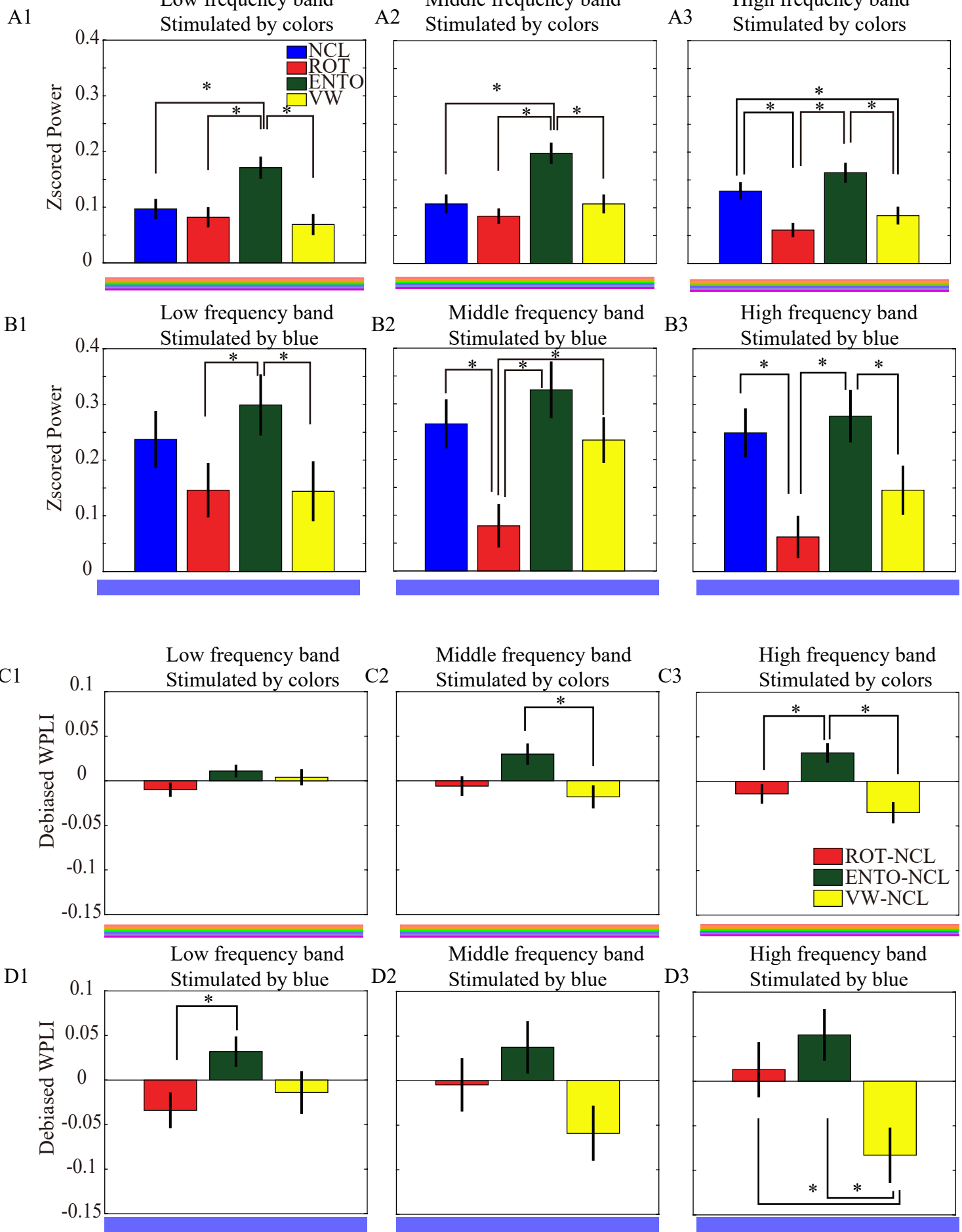

Supplement: Supplementary file 6 — Supplementary Figure 5. [file 41598_2020_76542_MOESM6_ESM.pdf]

A.

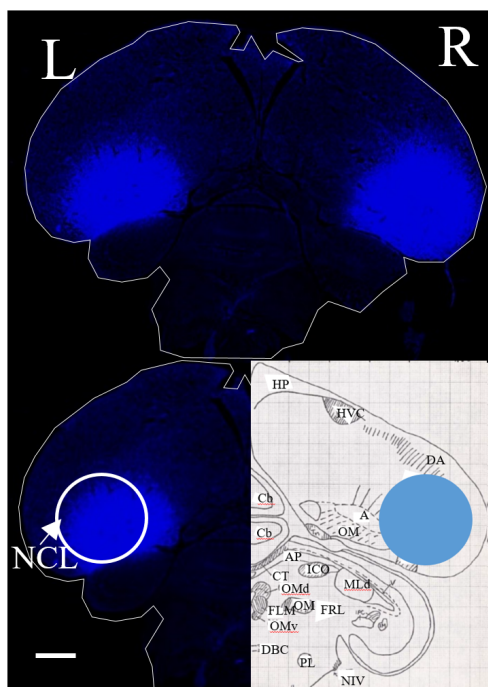

B.

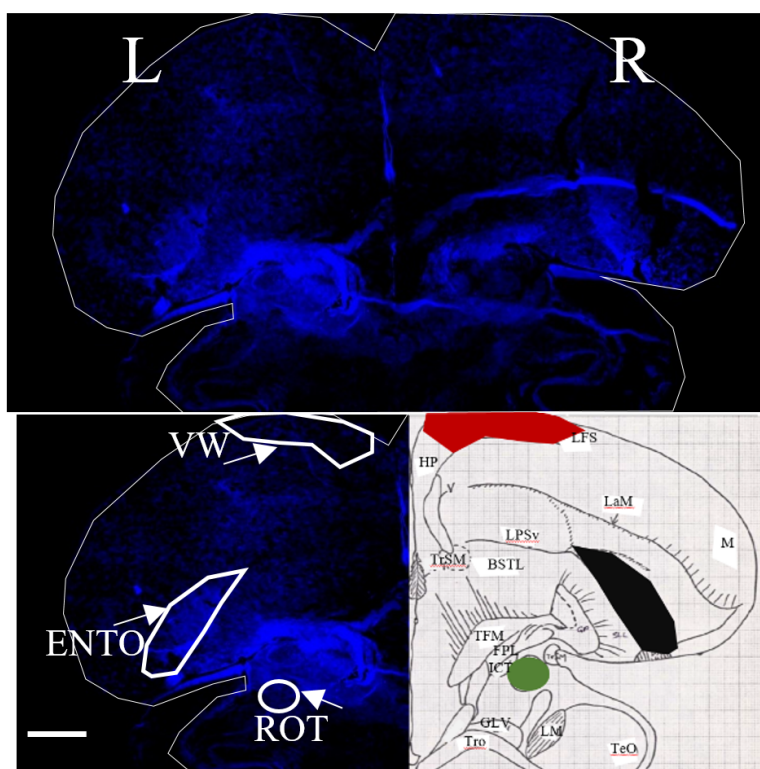

C.

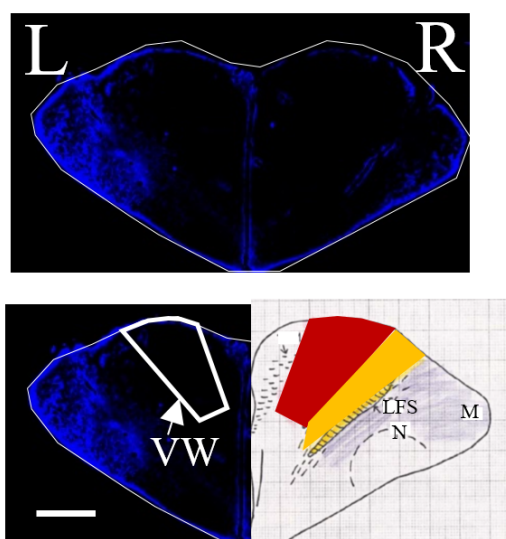

D.

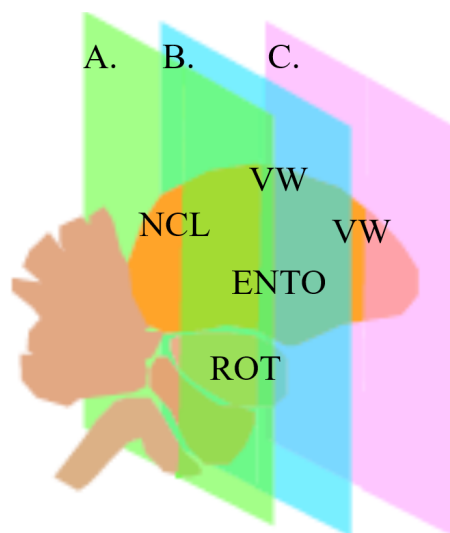

E.

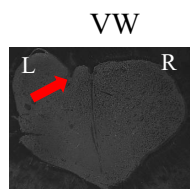

F.

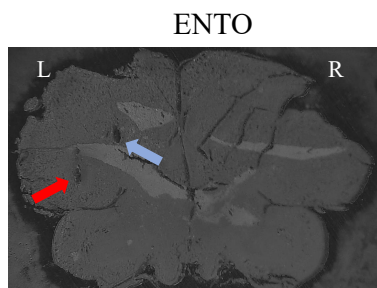

I.

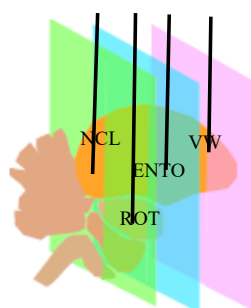

G.

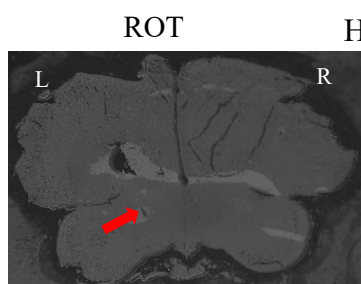

H.

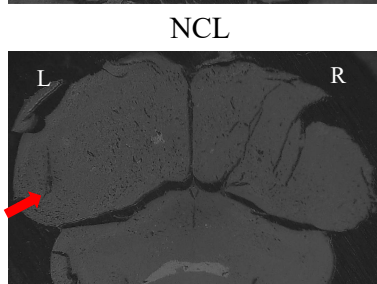

Supplement: Supplementary file 7 — Supplementary Figure 6. [file 41598_2020_76542_MOESM7_ESM.pdf]
