# Supplementary material for: Connectivity between nidopallium caudolateral and visual pathways in color perception of zebra finches
Source: Sci Rep. 2020 Nov 9;10:19382. doi: 10.1038/s41598-020-76542-z (PMC7653952; doi:10.1038/s41598-020-76542-z)
Supplement: Supplementary file 8 — Supplementary Figure 7. [file 41598_2020_76542_MOESM8_ESM.pdf]

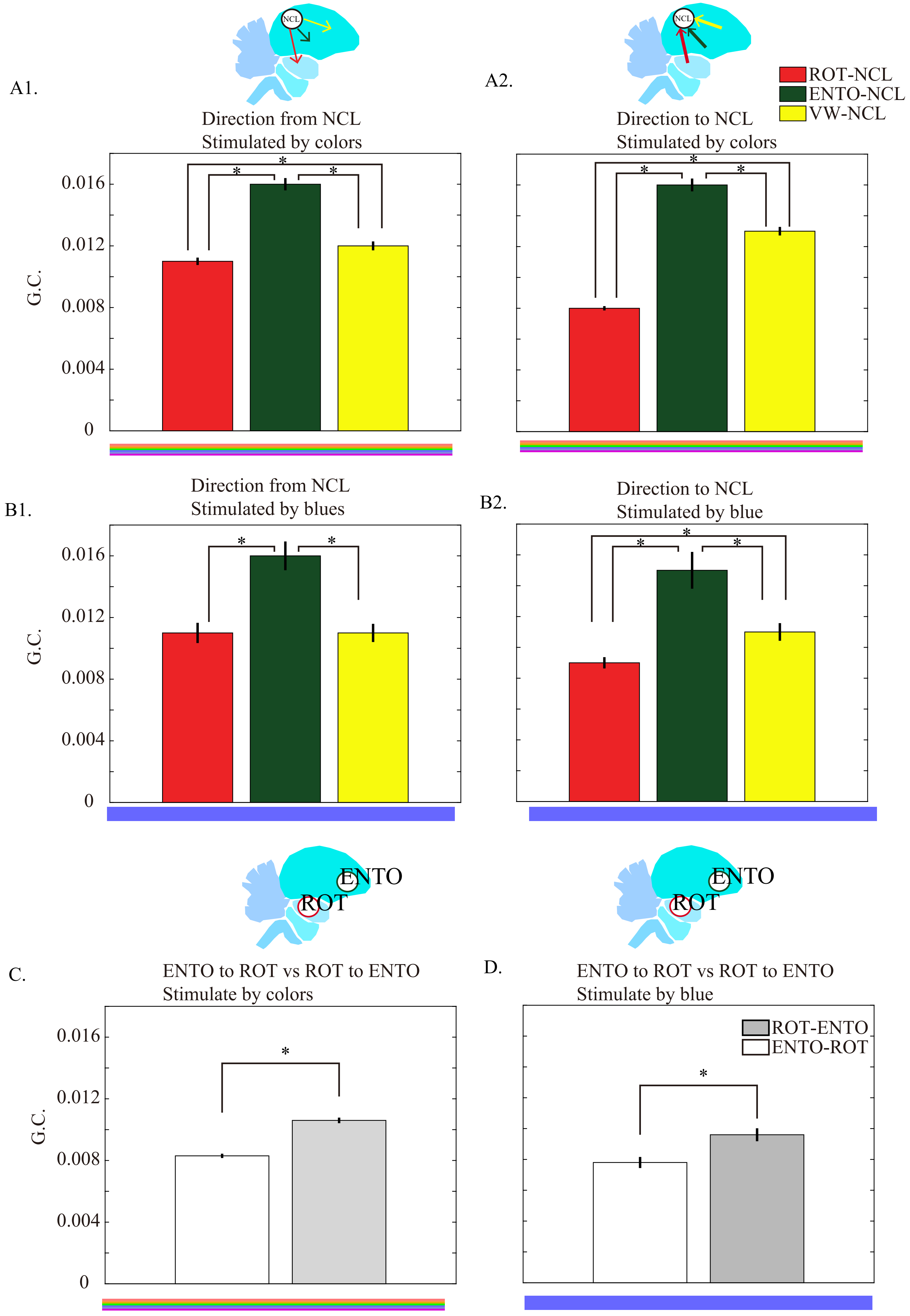

A1.
NCL
Direction from NCL
Stimulated by colors
A2.
NCL
Direction to NCL
Stimulated by colors
ROT-NCL
ENTO-NCL
VW-NCL
G.C.
0.016
0.012
0.008
0.004
0
*
B1.
Direction from NCL
Stimulated by blues
B2.
Direction to NCL
Stimulated by blue
ENTO
ROT
C.
ENTO to ROT vs ROT to ENTO
Stimulate by colors
D.
ENTO to ROT vs ROT to ENTO
Stimulate by blue
ROT-ENTO
ENTO-ROT
