# Supplementary material for: Connectivity between nidopallium caudolateral and visual pathways in color perception of zebra finches
Source: Sci Rep. 2020 Nov 9;10:19382. doi: 10.1038/s41598-020-76542-z (PMC7653952; doi:10.1038/s41598-020-76542-z)
Supplement: Supplementary file 9 — Supplementary Tables. [file 41598_2020_76542_MOESM9_ESM.docx]

|  | Color No. | VIS Radiation Power (Φe) | cd (I) | lx (E) | lumen (Φv) | RGB code |
| --- | --- | --- | --- | --- | --- | --- |
|  | 1 | 41.9 | 4.8 | 4.8 | 0.2 | (0, 0, 0.75) |
|  | 2 | 74.2 | 7.2 | 7.2 | 0.4 | (0, 0, 1) |
|  | 3 | 75.8 | 9.7 | 9.7 | 0.5 | (0, 0.25, 1) |
|  | 4 | 89.6 | 22.2 | 22.2 | 1.1 | (0, 0.5, 1) |
|  | 5 | 116.6 | 45.3 | 45.3 | 2.2 | (0, 0.75, 1) |
|  | 6 | 159.1 | 80.7 | 80.7 | 4.0 | (0, 1, 1) |
|  | 7 | 132.2 | 79.2 | 79.2 | 3.9 | (0.25, 1, 0.75) |
|  | 8 | 119.0 | 80.0 | 80.0 | 4.0 | (0.5, 1, 0.5) |
|  | 9 | 125.8 | 85.1 | 85.1 | 4.2 | (0.75, 1, 0.25) |
|  | 10 | 151.1 | 93.9 | 93.9 | 4.6 | (1, 1, 0) |
|  | 11 | 107.8 | 58.4 | 58.4 | 2.9 | (1, 0.75, 0) |
|  | 12 | 83.7 | 35.8 | 35.8 | 1.8 | (1, 0.5, 0) |
|  | 13 | 67.7 | 22.9 | 22.9 | 1.1 | (1, 0.25, 0) |
|  | 14 | 63.7 | 19.9 | 19.9 | 1.0 | (1, 0, 0) |
|  | 15 | 40.0 | 12.0 | 12.0 | 0.6 | (0.75, 0, 0) |
|  | Black | 10.4 | 2.4 | 2.4 | 0.1 | (0, 0, 0) |
|  | White | 211.7 | 97.5 | 97.5 | 4.8 | (1, 1, 1) |
|  | Rainbow1 | 87.9 | 33.0 | 33.0 | 1.6 | (1, 0.47, 0.47) |
|  | Rainbow2 | 93.0 | 41.5 | 41.5 | 2.1 | (1, 0.58, 0) |
|  | Rainbow3 | 88.7 | 46.6 | 46.6 | 2.3 | (0.86, 0.68, 0) |
|  | Rainbow4 | 93.9 | 74.1 | 74.1 | 3.7 | (0, 1, 0) |
|  | Rainbow5 | 90.7 | 17.8 | 17.8 | 0.9 | (0.4, 0.4, 1) |
|  | Rainbow6 | 92.8 | 27.0 | 27.0 | 1.3 | (0.51, 0.54, 0.87) |
|  | Rainbow7 | 88.2 | 17.7 | 17.7 | 0.9 | (0.85, 0, 0.85) |

Table S1 The intensities and RGB codes of different colors, black and white colors. We adjusted the rainbow colors to a similar VIS radiation power.

Table S2. One-way repeated measures ANOVA

F_low frequency (15, 304)_ = 7.84, p < 0.001

| Power NCL  Low Frequency  Corrected p values | | Color 1 | Color 2 | Color 3 | Color 4 | Color 5 | Color 6 | Color 7 | Color 8 | Color 9 | Color 10 | Color 11 | Color 12 | Color 13 | Color 14 | Color 15 |
| --- | --- | --- | --- | --- | --- | --- | --- | --- | --- | --- | --- | --- | --- | --- | --- | --- |
| Color 1 | |  |  |  |  |  |  |  |  |  |  |  |  |  |  |  |
| Color 2 | | 1.000 |  |  |  |  |  |  |  |  |  |  |  |  |  |  |
| Color 3 | | 1.000 | 1.000 |  |  |  |  |  |  |  |  |  |  |  |  |  |
| Color 4 | | 1.000 | 1.000 | 1.000 |  |  |  |  |  |  |  |  |  |  |  |  |
| Color 5 | | 1.000 | 1.000 | 1.000 | 1.000 |  |  |  |  |  |  |  |  |  |  |  |
| Color 6 | | 0.343 | 1.000 | 1.000 | 1.000 | 1.000 |  |  |  |  |  |  |  |  |  |  |
| Color 7 | | 0.022 | 1.000 | 1.000 | 1.000 | 1.000 | 1.000 |  |  |  |  |  |  |  |  |  |
| Color 8 | | 1.000 | 1.000 | 1.000 | 1.000 | 1.000 | 1.000 | 1.000 |  |  |  |  |  |  |  |  |
| Color 9 | | 1.000 | 1.000 | 1.000 | 1.000 | 1.000 | 1.000 | 1.000 | 1.000 |  |  |  |  |  |  |  |
| Color 10 | | 1.000 | 1.000 | 1.000 | 1.000 | 1.000 | 0.999 | 0.279 | 1.000 | 1.000 |  |  |  |  |  |  |
| Color 11 | | 1.000 | 1.000 | 1.000 | 1.000 | 1.000 | 1.000 | 1.000 | 1.000 | 1.000 | 1.000 |  |  |  |  |  |
| Color 12 | | 1.000 | 1.000 | 1.000 | 0.986 | 1.000 | 0.060 | 0.005 | 1.000 | 0.475 | 1.000 | 1.000 |  |  |  |  |
| Color 13 | | 1.000 | 1.000 | 1.000 | 1.000 | 1.000 | 1.000 | 1.000 | 1.000 | 1.000 | 1.000 | 1.000 | 1.000 |  |  |  |
| Color 14 | | 1.000 | 0.099 | 1.000 | 0.128 | 0.282 | 0.004 | < 0.001 | 1.000 | 0.053 | 1.000 | 0.157 | 1.000 | 0.694 |  |  |
| Color 15 | | 1.000 | 1.000 | 1.000 | 1.000 | 1.000 | 0.100 | 0.007 | 1.000 | 0.482 | 1.000 | 1.000 | 1.000 | 1.000 | 1.000 |  |
|  | | < 0.001 | < 0.001 | < 0.001 | < 0.001 | < 0.001 | < 0.001 | < 0.001 | < 0.001 | < 0.001 | < 0.001 | < 0.001 | 0.001 | < 0.001 | 0.011 | 0.002 |
|  |  |  |  |  |  |  |  |  |  |  |  |  |  |  |  |  |
| Mean | -0.069 | 0.141 | 0.225 | 0.195 | 0.224 | 0.221 | 0.264 | 0.290 | 0.198 | 0.235 | 0.160 | 0.222 | 0.116 | 0.205 | 0.090 | 0.116 |
| SEM | 0.025 | 0.032 | 0.033 | 0.030 | 0.030 | 0.031 | 0.031 | 0.032 | 0.032 | 0.032 | 0.032 | 0.033 | 0.032 | 0.034 | 0.032 | 0.033 |

Table S2 cont.

F_middle frequency (15, 304)_ = 9.79, p < 0.001

| Power NCL  Middle Frequency  Corrected p values | | Color 1 | Color 2 | Color 3 | Color 4 | Color 5 | Color 6 | Color 7 | Color 8 | Color 9 | Color 10 | Color 11 | Color 12 | Color 13 | Color 14 | Color 15 |
| --- | --- | --- | --- | --- | --- | --- | --- | --- | --- | --- | --- | --- | --- | --- | --- | --- |
| Color 1 | |  |  |  |  |  |  |  |  |  |  |  |  |  |  |  |
| Color 2 | | 1.000 |  |  |  |  |  |  |  |  |  |  |  |  |  |  |
| Color 3 | | 1.000 | 1.000 |  |  |  |  |  |  |  |  |  |  |  |  |  |
| Color 4 | | 1.000 | 1.000 | 1.000 |  |  |  |  |  |  |  |  |  |  |  |  |
| Color 5 | | 1.000 | 1.000 | 1.000 | 1.000 |  |  |  |  |  |  |  |  |  |  |  |
| Color 6 | | 0.016 | 1.000 | 1.000 | 0.275 | 1.000 |  |  |  |  |  |  |  |  |  |  |
| Color 7 | | 0.001 | 0.977 | 0.196 | 0.048 | 0.907 | 1.000 |  |  |  |  |  |  |  |  |  |
| Color 8 | | 1.000 | 1.000 | 1.000 | 1.000 | 1.000 | 0.823 | 0.096 |  |  |  |  |  |  |  |  |
| Color 9 | | 0.259 | 1.000 | 1.000 | 1.000 | 1.000 | 1.000 | 1.000 | 1.000 |  |  |  |  |  |  |  |
| Color 10 | | 1.000 | 1.000 | 1.000 | 1.000 | 1.000 | 0.056 | 0.009 | 1.000 | 0.505 |  |  |  |  |  |  |
| Color 11 | | 1.000 | 1.000 | 1.000 | 1.000 | 1.000 | 1.000 | 1.000 | 1.000 | 1.000 | 1.000 |  |  |  |  |  |
| Color 12 | | 1.000 | 1.000 | 1.000 | 1.000 | 0.713 | < 0.001 | < 0.001 | 1.000 | 0.011 | 1.000 | 0.060 |  |  |  |  |
| Color 13 | | 1.000 | 1.000 | 1.000 | 1.000 | 1.000 | 1.000 | 0.844 | 1.000 | 1.000 | 1.000 | 1.000 | 0.384 |  |  |  |
| Color 14 | | 1.000 | 0.020 | 0.036 | 0.327 | 0.012 | < 0.001 | < 0.001 | 0.137 | < 0.001 | 1.000 | 0.001 | 1.000 | 0.008 |  |  |
| Color 15 | | 1.000 | 1.000 | 1.000 | 1.000 | 0.719 | 0.002 | < 0.001 | 1.000 | 0.044 | 1.000 | 0.268 | 1.000 | 0.726 | 1.000 |  |
|  | | < 0.001 | < 0.001 | < 0.001 | < 0.001 | < 0.001 | < 0.001 | < 0.001 | < 0.001 | < 0.001 | < 0.001 | < 0.001 | < 0.001 | < 0.001 | 0.021 | 0.001 |
|  |  |  |  |  |  |  |  |  |  |  |  |  |  |  |  |  |
| Mean | -0.055 | 0.166 | 0.235 | 0.229 | 0.203 | 0.241 | 0.321 | 0.343 | 0.220 | 0.288 | 0.179 | 0.266 | 0.131 | 0.241 | 0.086 | 0.139 |
| SEM | 0.024 | 0.032 | 0.032 | 0.030 | 0.029 | 0.030 | 0.031 | 0.033 | 0.032 | 0.032 | 0.032 | 0.034 | 0.031 | 0.032 | 0.030 | 0.032 |

Table S2 cont.

F_high frequency (15, 304)_ = 11.52, p < 0.001

| Power NCL  High Frequency  Corrected p values | | Color 1 | Color 2 | Color 3 | Color 4 | Color 5 | Color 6 | Color 7 | Color 8 | Color 9 | Color 10 | Color 11 | Color 12 | Color 13 | Color 14 | Color 15 |
| --- | --- | --- | --- | --- | --- | --- | --- | --- | --- | --- | --- | --- | --- | --- | --- | --- |
| Color 1 | |  |  |  |  |  |  |  |  |  |  |  |  |  |  |  |
| Color 2 | | 1.000 |  |  |  |  |  |  |  |  |  |  |  |  |  |  |
| Color 3 | | 1.000 | 1.000 |  |  |  |  |  |  |  |  |  |  |  |  |  |
| Color 4 | | 1.000 | 1.000 | 1.000 |  |  |  |  |  |  |  |  |  |  |  |  |
| Color 5 | | 1.000 | 1.000 | 1.000 | 1.000 |  |  |  |  |  |  |  |  |  |  |  |
| Color 6 | | 0.626 | 1.000 | 1.000 | 1.000 | 1.000 |  |  |  |  |  |  |  |  |  |  |
| Color 7 | | 0.008 | 1.000 | 0.096 | 0.050 | 0.111 | 1.000 |  |  |  |  |  |  |  |  |  |
| Color 8 | | 1.000 | 1.000 | 1.000 | 1.000 | 1.000 | 1.000 | 0.155 |  |  |  |  |  |  |  |  |
| Color 9 | | 0.280 | 1.000 | 1.000 | 0.875 | 1.000 | 1.000 | 1.000 | 1.000 |  |  |  |  |  |  |  |
| Color 10 | | 1.000 | 1.000 | 1.000 | 1.000 | 1.000 | 0.208 | 0.002 | 1.000 | 0.084 |  |  |  |  |  |  |
| Color 11 | | 1.000 | 1.000 | 1.000 | 1.000 | 1.000 | 1.000 | 1.000 | 1.000 | 1.000 | 1.000 |  |  |  |  |  |
| Color 12 | | 1.000 | 0.357 | 1.000 | 1.000 | 1.000 | 0.003 | < 0.001 | 1.000 | 0.002 | 1.000 | 0.049 |  |  |  |  |
| Color 13 | | 1.000 | 1.000 | 1.000 | 1.000 | 1.000 | 1.000 | 0.127 | 1.000 | 1.000 | 1.000 | 1.000 | 1.000 |  |  |  |
| Color 14 | | 1.000 | 0.025 | 0.211 | 1.000 | 0.239 | < 0.001 | < 0.001 | 0.620 | < 0.001 | 1.000 | 0.002 | 1.000 | 0.455 |  |  |
| Color 15 | | 1.000 | 0.571 | 1.000 | 1.000 | 1.000 | 0.008 | < 0.001 | 1.000 | 0.004 | 1.000 | 0.062 | 1.000 | 1.000 | 1.000 |  |
|  | | < 0.001 | < 0.001 | < 0.001 | < 0.001 | < 0.001 | < 0.001 | < 0.001 | < 0.001 | < 0.001 | < 0.001 | < 0.001 | < 0.001 | < 0.001 | < 0.001 | < 0.001 |
|  |  |  |  |  |  |  |  |  |  |  |  |  |  |  |  |  |
| Mean | -0.089 | 0.161 | 0.222 | 0.197 | 0.178 | 0.196 | 0.266 | 0.309 | 0.194 | 0.273 | 0.148 | 0.243 | 0.108 | 0.192 | 0.085 | 0.113 |
| SEM | 0.022 | 0.028 | 0.029 | 0.027 | 0.025 | 0.027 | 0.028 | 0.030 | 0.029 | 0.029 | 0.030 | 0.031 | 0.029 | 0.030 | 0.027 | 0.029 |

Table S2 cont.

F_low frequency (15, 304)_ = 6.93, p < 0.001

| Power ROT  Low Frequency  Corrected p values | | Color 1 | Color 2 | Color 3 | Color 4 | Color 5 | Color 6 | Color 7 | Color 8 | Color 9 | Color 10 | Color 11 | Color 12 | Color 13 | Color 14 | Color 15 |
| --- | --- | --- | --- | --- | --- | --- | --- | --- | --- | --- | --- | --- | --- | --- | --- | --- |
| Color 1 | |  |  |  |  |  |  |  |  |  |  |  |  |  |  |  |
| Color 2 | | 0.601 |  |  |  |  |  |  |  |  |  |  |  |  |  |  |
| Color 3 | | 1.000 | 1.000 |  |  |  |  |  |  |  |  |  |  |  |  |  |
| Color 4 | | 1.000 | 1.000 | 1.000 |  |  |  |  |  |  |  |  |  |  |  |  |
| Color 5 | | 1.000 | 1.000 | 1.000 | 1.000 |  |  |  |  |  |  |  |  |  |  |  |
| Color 6 | | 0.035 | 1.000 | 1.000 | 1.000 | 1.000 |  |  |  |  |  |  |  |  |  |  |
| Color 7 | | 0.051 | 1.000 | 1.000 | 1.000 | 1.000 | 1.000 |  |  |  |  |  |  |  |  |  |
| Color 8 | | 0.742 | 1.000 | 1.000 | 1.000 | 1.000 | 1.000 | 1.000 |  |  |  |  |  |  |  |  |
| Color 9 | | 1.000 | 1.000 | 1.000 | 1.000 | 1.000 | 1.000 | 1.000 | 1.000 |  |  |  |  |  |  |  |
| Color 10 | | 1.000 | 1.000 | 1.000 | 1.000 | 1.000 | 1.000 | 1.000 | 1.000 | 1.000 |  |  |  |  |  |  |
| Color 11 | | 1.000 | 1.000 | 1.000 | 1.000 | 1.000 | 1.000 | 1.000 | 1.000 | 1.000 | 1.000 |  |  |  |  |  |
| Color 12 | | 1.000 | 1.000 | 1.000 | 1.000 | 1.000 | 0.140 | 0.173 | 1.000 | 1.000 | 1.000 | 1.000 |  |  |  |  |
| Color 13 | | 0.082 | 1.000 | 1.000 | 1.000 | 1.000 | 1.000 | 1.000 | 1.000 | 1.000 | 1.000 | 1.000 | 0.183 |  |  |  |
| Color 14 | | 1.000 | 0.523 | 1.000 | 1.000 | 1.000 | 0.049 | 0.116 | 1.000 | 1.000 | 1.000 | 1.000 | 1.000 | 0.152 |  |  |
| Color 15 | | 1.000 | 1.000 | 1.000 | 1.000 | 1.000 | 1.000 | 1.000 | 1.000 | 1.000 | 1.000 | 1.000 | 1.000 | 1.000 | 1.000 |  |
|  | | 0.003 | < 0.001 | < 0.001 | < 0.001 | < 0.001 | < 0.001 | < 0.001 | < 0.001 | < 0.001 | < 0.001 | < 0.001 | 0.003 | < 0.001 | 0.003 | < 0.001 |
|  |  |  |  |  |  |  |  |  |  |  |  |  |  |  |  |  |
| Mean | -0.095 | 0.069 | 0.187 | 0.141 | 0.148 | 0.143 | 0.206 | 0.204 | 0.172 | 0.164 | 0.128 | 0.167 | 0.076 | 0.201 | 0.068 | 0.116 |
| SEM | 0.024 | 0.030 | 0.032 | 0.031 | 0.028 | 0.030 | 0.031 | 0.032 | 0.032 | 0.031 | 0.031 | 0.033 | 0.032 | 0.032 | 0.030 | 0.032 |

Table S2 cont.

F_middle frequency (15, 304)_ = 12.42, p < 0.001

| Power ROT  Middle Frequency  Corrected p values | | Color 1 | Color 2 | Color 3 | Color 4 | Color 5 | Color 6 | Color 7 | Color 8 | Color 9 | Color 10 | Color 11 | Color 12 | Color 13 | Color 14 | Color 15 |
| --- | --- | --- | --- | --- | --- | --- | --- | --- | --- | --- | --- | --- | --- | --- | --- | --- |
| Color 1 | |  |  |  |  |  |  |  |  |  |  |  |  |  |  |  |
| Color 2 | | 1.000 |  |  |  |  |  |  |  |  |  |  |  |  |  |  |
| Color 3 | | 1.000 | 1.000 |  |  |  |  |  |  |  |  |  |  |  |  |  |
| Color 4 | | 1.000 | 1.000 | 1.000 |  |  |  |  |  |  |  |  |  |  |  |  |
| Color 5 | | 1.000 | 1.000 | 1.000 | 1.000 |  |  |  |  |  |  |  |  |  |  |  |
| Color 6 | | < 0.001 | 0.231 | 0.010 | 0.003 | 1.000 |  |  |  |  |  |  |  |  |  |  |
| Color 7 | | < 0.001 | 0.012 | < 0.001 | < 0.001 | 0.072 | 1.000 |  |  |  |  |  |  |  |  |  |
| Color 8 | | 0.057 | 1.000 | 1.000 | 0.623 | 1.000 | 1.000 | 0.779 |  |  |  |  |  |  |  |  |
| Color 9 | | < 0.001 | 0.169 | 0.004 | 0.001 | 1.000 | 1.000 | 1.000 | 1.000 |  |  |  |  |  |  |  |
| Color 10 | | 1.000 | 1.000 | 1.000 | 1.000 | 1.000 | 0.062 | 0.002 | 1.000 | 0.012 |  |  |  |  |  |  |
| Color 11 | | 0.233 | 1.000 | 1.000 | 0.950 | 1.000 | 1.000 | 0.640 | 1.000 | 1.000 | 1.000 |  |  |  |  |  |
| Color 12 | | 1.000 | 1.000 | 1.000 | 1.000 | 1.000 | 0.171 | 0.001 | 1.000 | 0.041 | 1.000 | 1.000 |  |  |  |  |
| Color 13 | | < 0.001 | 0.028 | 0.001 | < 0.001 | 0.545 | 1.000 | 1.000 | 1.000 | 1.000 | 0.011 | 1.000 | 0.003 |  |  |  |
| Color 14 | | 1.000 | 1.000 | 1.000 | 1.000 | 0.054 | < 0.001 | < 0.001 | 0.003 | < 0.001 | 1.000 | 0.011 | 1.000 | < 0.001 |  |  |
| Color 15 | | 1.000 | 1.000 | 1.000 | 1.000 | 1.000 | 0.162 | 0.002 | 1.000 | 0.034 | 1.000 | 1.000 | 1.000 | 0.011 | 1.000 |  |
|  | | < 0.001 | < 0.001 | < 0.001 | < 0.001 | < 0.001 | < 0.001 | < 0.001 | < 0.001 | < 0.001 | < 0.001 | < 0.001 | < 0.001 | < 0.001 | 0.001 | < 0.001 |
|  |  |  |  |  |  |  |  |  |  |  |  |  |  |  |  |  |
| Mean | -0.084 | 0.105 | 0.158 | 0.139 | 0.120 | 0.191 | 0.263 | 0.304 | 0.219 | 0.273 | 0.150 | 0.211 | 0.155 | 0.292 | 0.071 | 0.149 |
| SEM | 0.021 | 0.029 | 0.030 | 0.028 | 0.025 | 0.027 | 0.028 | 0.030 | 0.030 | 0.028 | 0.030 | 0.031 | 0.029 | 0.031 | 0.028 | 0.031 |

Table S2 cont.

F_high frequency (15, 304)_ = 7.31, p < 0.001

| Power ROT High Frequency  Corrected p values | | Color 1 | Color 2 | Color 3 | Color 4 | Color 5 | Color 6 | Color 7 | Color 8 | Color 9 | Color 10 | Color 11 | Color 12 | Color 13 | Color 14 | Color 15 |
| --- | --- | --- | --- | --- | --- | --- | --- | --- | --- | --- | --- | --- | --- | --- | --- | --- |
| Color 1 | |  |  |  |  |  |  |  |  |  |  |  |  |  |  |  |
| Color 2 | | 1.000 |  |  |  |  |  |  |  |  |  |  |  |  |  |  |
| Color 3 | | 1.000 | 1.000 |  |  |  |  |  |  |  |  |  |  |  |  |  |
| Color 4 | | 1.000 | 1.000 | 1.000 |  |  |  |  |  |  |  |  |  |  |  |  |
| Color 5 | | 1.000 | 1.000 | 1.000 | 1.000 |  |  |  |  |  |  |  |  |  |  |  |
| Color 6 | | 0.431 | 1.000 | 1.000 | 1.000 | 1.000 |  |  |  |  |  |  |  |  |  |  |
| Color 7 | | 0.023 | 1.000 | 1.000 | 0.121 | 0.217 | 1.000 |  |  |  |  |  |  |  |  |  |
| Color 8 | | 1.000 | 1.000 | 1.000 | 1.000 | 1.000 | 1.000 | 1.000 |  |  |  |  |  |  |  |  |
| Color 9 | | 0.103 | 1.000 | 1.000 | 0.213 | 0.523 | 1.000 | 1.000 | 1.000 |  |  |  |  |  |  |  |
| Color 10 | | 1.000 | 1.000 | 1.000 | 1.000 | 1.000 | 1.000 | 0.202 | 1.000 | 0.327 |  |  |  |  |  |  |
| Color 11 | | 1.000 | 1.000 | 1.000 | 1.000 | 1.000 | 1.000 | 1.000 | 1.000 | 1.000 | 1.000 |  |  |  |  |  |
| Color 12 | | 1.000 | 1.000 | 1.000 | 1.000 | 1.000 | 0.506 | 0.013 | 1.000 | 0.072 | 1.000 | 1.000 |  |  |  |  |
| Color 13 | | 0.574 | 1.000 | 1.000 | 1.000 | 1.000 | 1.000 | 1.000 | 1.000 | 1.000 | 1.000 | 1.000 | 0.895 |  |  |  |
| Color 14 | | 1.000 | 1.000 | 1.000 | 1.000 | 1.000 | 0.006 | < 0.001 | 0.325 | 0.001 | 1.000 | 0.087 | 1.000 | 0.011 |  |  |
| Color 15 | | 1.000 | 1.000 | 1.000 | 1.000 | 1.000 | 1.000 | 0.695 | 1.000 | 1.000 | 1.000 | 1.000 | 1.000 | 1.000 | 1.000 |  |
|  | | < 0.001 | < 0.001 | < 0.001 | < 0.001 | < 0.001 | < 0.001 | < 0.001 | < 0.001 | < 0.001 | < 0.001 | < 0.001 | < 0.001 | < 0.001 | < 0.001 | < 0.001 |
|  |  |  |  |  |  |  |  |  |  |  |  |  |  |  |  |  |
| Mean | -0.080 | 0.108 | 0.139 | 0.153 | 0.114 | 0.129 | 0.196 | 0.224 | 0.162 | 0.218 | 0.123 | 0.173 | 0.112 | 0.194 | 0.068 | 0.136 |
| SEM | 0.020 | 0.026 | 0.026 | 0.027 | 0.023 | 0.025 | 0.027 | 0.028 | 0.028 | 0.027 | 0.028 | 0.029 | 0.027 | 0.029 | 0.023 | 0.027 |

Table S2 cont.

F_low frequency (15, 304)_ = 7.49, p < 0.001

| Power ENTO  Low Frequency  Corrected p values | | Color 1 | Color 2 | Color 3 | Color 4 | Color 5 | Color 6 | Color 7 | Color 8 | Color 9 | Color 10 | Color 11 | Color 12 | Color 13 | Color 14 | Color 15 |
| --- | --- | --- | --- | --- | --- | --- | --- | --- | --- | --- | --- | --- | --- | --- | --- | --- |
| Color 1 | |  |  |  |  |  |  |  |  |  |  |  |  |  |  |  |
| Color 2 | | 1.000 |  |  |  |  |  |  |  |  |  |  |  |  |  |  |
| Color 3 | | 1.000 | 1.000 |  |  |  |  |  |  |  |  |  |  |  |  |  |
| Color 4 | | 1.000 | 1.000 | 1.000 |  |  |  |  |  |  |  |  |  |  |  |  |
| Color 5 | | 1.000 | 1.000 | 1.000 | 1.000 |  |  |  |  |  |  |  |  |  |  |  |
| Color 6 | | 0.303 | 1.000 | 1.000 | 1.000 | 1.000 |  |  |  |  |  |  |  |  |  |  |
| Color 7 | | 0.002 | 1.000 | 1.000 | 0.077 | 1.000 | 1.000 |  |  |  |  |  |  |  |  |  |
| Color 8 | | 1.000 | 1.000 | 1.000 | 1.000 | 1.000 | 1.000 | 0.038 |  |  |  |  |  |  |  |  |
| Color 9 | | 0.109 | 1.000 | 1.000 | 1.000 | 1.000 | 1.000 | 1.000 | 0.571 |  |  |  |  |  |  |  |
| Color 10 | | 1.000 | 1.000 | 1.000 | 1.000 | 1.000 | 1.000 | 0.239 | 1.000 | 1.000 |  |  |  |  |  |  |
| Color 11 | | 1.000 | 1.000 | 1.000 | 1.000 | 1.000 | 1.000 | 1.000 | 1.000 | 1.000 | 1.000 |  |  |  |  |  |
| Color 12 | | 1.000 | 1.000 | 1.000 | 1.000 | 1.000 | 0.148 | 0.002 | 1.000 | 0.023 | 1.000 | 1.000 |  |  |  |  |
| Color 13 | | 1.000 | 1.000 | 1.000 | 1.000 | 1.000 | 1.000 | 1.000 | 1.000 | 1.000 | 1.000 | 1.000 | 1.000 |  |  |  |
| Color 14 | | 1.000 | 1.000 | 0.427 | 1.000 | 0.327 | 0.016 | < 0.001 | 1.000 | 0.003 | 1.000 | 0.565 | 1.000 | 0.912 |  |  |
| Color 15 | | 1.000 | 1.000 | 1.000 | 1.000 | 1.000 | 1.000 | 0.023 | 1.000 | 0.343 | 1.000 | 1.000 | 1.000 | 1.000 | 1.000 |  |
|  | | < 0.001 | < 0.001 | < 0.001 | < 0.001 | < 0.001 | < 0.001 | < 0.001 | < 0.001 | < 0.001 | < 0.001 | < 0.001 | 0.001 | < 0.001 | 0.005 | < 0.001 |
|  |  |  |  |  |  |  |  |  |  |  |  |  |  |  |  |  |
| Mean | -0.075 | 0.144 | 0.216 | 0.234 | 0.186 | 0.239 | 0.279 | 0.331 | 0.179 | 0.292 | 0.195 | 0.229 | 0.129 | 0.224 | 0.100 | 0.160 |
| SEM | 0.027 | 0.034 | 0.034 | 0.033 | 0.031 | 0.034 | 0.035 | 0.036 | 0.034 | 0.034 | 0.033 | 0.036 | 0.036 | 0.036 | 0.033 | 0.035 |

Table S2 cont.

F_middle frequency (15, 304)_ = 9.75, p < 0.001

| Power ENTO  Middle Frequency  Corrected p values | | Color 1 | Color 2 | Color 3 | Color 4 | Color 5 | Color 6 | Color 7 | Color 8 | Color 9 | Color 10 | Color 11 | Color 12 | Color 13 | Color 14 | Color 15 |
| --- | --- | --- | --- | --- | --- | --- | --- | --- | --- | --- | --- | --- | --- | --- | --- | --- |
| Color 1 | |  |  |  |  |  |  |  |  |  |  |  |  |  |  |  |
| Color 2 | | 1.000 |  |  |  |  |  |  |  |  |  |  |  |  |  |  |
| Color 3 | | 1.000 | 1.000 |  |  |  |  |  |  |  |  |  |  |  |  |  |
| Color 4 | | 1.000 | 1.000 | 1.000 |  |  |  |  |  |  |  |  |  |  |  |  |
| Color 5 | | 1.000 | 1.000 | 1.000 | 1.000 |  |  |  |  |  |  |  |  |  |  |  |
| Color 6 | | 0.044 | 1.000 | 1.000 | 0.019 | 1.000 |  |  |  |  |  |  |  |  |  |  |
| Color 7 | | 0.005 | 1.000 | 1.000 | 0.004 | 1.000 | 1.000 |  |  |  |  |  |  |  |  |  |
| Color 8 | | 1.000 | 1.000 | 1.000 | 1.000 | 1.000 | 1.000 | 1.000 |  |  |  |  |  |  |  |  |
| Color 9 | | 0.220 | 1.000 | 1.000 | 0.100 | 1.000 | 1.000 | 1.000 | 1.000 |  |  |  |  |  |  |  |
| Color 10 | | 1.000 | 1.000 | 1.000 | 1.000 | 0.925 | 0.039 | 0.006 | 1.000 | 0.071 |  |  |  |  |  |  |
| Color 11 | | 1.000 | 1.000 | 1.000 | 1.000 | 1.000 | 1.000 | 1.000 | 1.000 | 1.000 | 1.000 |  |  |  |  |  |
| Color 12 | | 1.000 | 1.000 | 1.000 | 1.000 | 0.465 | 0.012 | 0.001 | 1.000 | 0.034 | 1.000 | 0.481 |  |  |  |  |
| Color 13 | | 1.000 | 1.000 | 1.000 | 1.000 | 1.000 | 1.000 | 1.000 | 1.000 | 1.000 | 1.000 | 1.000 | 1.000 |  |  |  |
| Color 14 | | 1.000 | 0.169 | 0.059 | 1.000 | 0.007 | < 0.001 | < 0.001 | 0.094 | 0.001 | 1.000 | 0.005 | 1.000 | 0.052 |  |  |
| Color 15 | | 1.000 | 1.000 | 1.000 | 1.000 | 0.551 | 0.026 | 0.003 | 1.000 | 0.073 | 1.000 | 0.781 | 1.000 | 1.000 | 1.000 |  |
|  | | < 0.001 | < 0.001 | < 0.001 | < 0.001 | < 0.001 | < 0.001 | < 0.001 | < 0.001 | < 0.001 | < 0.001 | < 0.001 | < 0.001 | < 0.001 | 0.001 | < 0.001 |
|  |  |  |  |  |  |  |  |  |  |  |  |  |  |  |  |  |
| Mean | -0.059 | 0.192 | 0.254 | 0.272 | 0.184 | 0.295 | 0.341 | 0.359 | 0.262 | 0.322 | 0.185 | 0.289 | 0.167 | 0.266 | 0.119 | 0.178 |
| SEM | 0.026 | 0.032 | 0.033 | 0.032 | 0.030 | 0.033 | 0.033 | 0.034 | 0.033 | 0.033 | 0.033 | 0.035 | 0.034 | 0.034 | 0.031 | 0.034 |

Table S2 cont.

F_high frequency (15, 304)_ = 9.48, p < 0.001

| Power ENTO  High Frequency  Corrected p values | | Color 1 | Color 2 | Color 3 | Color 4 | Color 5 | Color 6 | Color 7 | Color 8 | Color 9 | Color 10 | Color 11 | Color 12 | Color 13 | Color 14 | Color 15 |
| --- | --- | --- | --- | --- | --- | --- | --- | --- | --- | --- | --- | --- | --- | --- | --- | --- |
| Color 1 | |  |  |  |  |  |  |  |  |  |  |  |  |  |  |  |
| Color 2 | | 1.000 |  |  |  |  |  |  |  |  |  |  |  |  |  |  |
| Color 3 | | 1.000 | 1.000 |  |  |  |  |  |  |  |  |  |  |  |  |  |
| Color 4 | | 1.000 | 1.000 | 1.000 |  |  |  |  |  |  |  |  |  |  |  |  |
| Color 5 | | 1.000 | 1.000 | 1.000 | 1.000 |  |  |  |  |  |  |  |  |  |  |  |
| Color 6 | | 1.000 | 1.000 | 1.000 | 1.000 | 1.000 |  |  |  |  |  |  |  |  |  |  |
| Color 7 | | 0.003 | 1.000 | 0.337 | 0.049 | 1.000 | 1.000 |  |  |  |  |  |  |  |  |  |
| Color 8 | | 1.000 | 1.000 | 1.000 | 1.000 | 1.000 | 1.000 | 1.000 |  |  |  |  |  |  |  |  |
| Color 9 | | 1.000 | 1.000 | 1.000 | 1.000 | 1.000 | 1.000 | 1.000 | 1.000 |  |  |  |  |  |  |  |
| Color 10 | | 1.000 | 1.000 | 1.000 | 1.000 | 1.000 | 1.000 | 0.046 | 1.000 | 1.000 |  |  |  |  |  |  |
| Color 11 | | 1.000 | 1.000 | 1.000 | 1.000 | 1.000 | 1.000 | 1.000 | 1.000 | 1.000 | 1.000 |  |  |  |  |  |
| Color 12 | | 1.000 | 1.000 | 1.000 | 1.000 | 0.401 | 0.561 | < 0.001 | 1.000 | 0.275 | 1.000 | 0.320 |  |  |  |  |
| Color 13 | | 1.000 | 1.000 | 1.000 | 1.000 | 1.000 | 1.000 | 1.000 | 1.000 | 1.000 | 1.000 | 1.000 | 1.000 |  |  |  |
| Color 14 | | 1.000 | 0.095 | 1.000 | 1.000 | 0.037 | 0.038 | < 0.001 | 0.339 | 0.019 | 1.000 | 0.015 | 1.000 | 0.483 |  |  |
| Color 15 | | 1.000 | 0.499 | 1.000 | 1.000 | 0.116 | 0.154 | < 0.001 | 1.000 | 0.062 | 1.000 | 0.064 | 1.000 | 1.000 | 1.000 |  |
|  | | < 0.001 | < 0.001 | < 0.001 | < 0.001 | < 0.001 | < 0.001 | < 0.001 | < 0.001 | < 0.001 | < 0.001 | < 0.001 | < 0.001 | < 0.001 | < 0.001 | < 0.001 |
|  |  |  |  |  |  |  |  |  |  |  |  |  |  |  |  |  |
| Mean | -0.067 | 0.173 | 0.255 | 0.223 | 0.195 | 0.264 | 0.265 | 0.332 | 0.243 | 0.271 | 0.186 | 0.272 | 0.153 | 0.235 | 0.121 | 0.139 |
| SEM | 0.025 | 0.030 | 0.030 | 0.030 | 0.028 | 0.029 | 0.030 | 0.031 | 0.030 | 0.030 | 0.031 | 0.032 | 0.031 | 0.031 | 0.029 | 0.031 |

Table S2 cont.

F_low frequency (15, 220)_ = 5.44, p < 0.001

| Power VW  Low Frequency  Corrected p values | | Color 1 | Color 2 | Color 3 | Color 4 | Color 5 | Color 6 | Color 7 | Color 8 | Color 9 | Color 10 | Color 11 | Color 12 | Color 13 | Color 14 | Color 15 |
| --- | --- | --- | --- | --- | --- | --- | --- | --- | --- | --- | --- | --- | --- | --- | --- | --- |
| Color 1 | |  |  |  |  |  |  |  |  |  |  |  |  |  |  |  |
| Color 2 | | 1.000 |  |  |  |  |  |  |  |  |  |  |  |  |  |  |
| Color 3 | | 1.000 | 1.000 |  |  |  |  |  |  |  |  |  |  |  |  |  |
| Color 4 | | 1.000 | 1.000 | 1.000 |  |  |  |  |  |  |  |  |  |  |  |  |
| Color 5 | | 1.000 | 1.000 | 1.000 | 1.000 |  |  |  |  |  |  |  |  |  |  |  |
| Color 6 | | 1.000 | 1.000 | 1.000 | 1.000 | 1.000 |  |  |  |  |  |  |  |  |  |  |
| Color 7 | | 1.000 | 1.000 | 1.000 | 1.000 | 1.000 | 1.000 |  |  |  |  |  |  |  |  |  |
| Color 8 | | 1.000 | 1.000 | 1.000 | 1.000 | 1.000 | 1.000 | 1.000 |  |  |  |  |  |  |  |  |
| Color 9 | | 1.000 | 1.000 | 1.000 | 1.000 | 1.000 | 1.000 | 1.000 | 1.000 |  |  |  |  |  |  |  |
| Color 10 | | 1.000 | 1.000 | 1.000 | 1.000 | 1.000 | 1.000 | 1.000 | 1.000 | 1.000 |  |  |  |  |  |  |
| Color 11 | | 0.570 | 1.000 | 1.000 | 1.000 | 1.000 | 1.000 | 1.000 | 0.604 | 1.000 | 1.000 |  |  |  |  |  |
| Color 12 | | 1.000 | 1.000 | 0.739 | 1.000 | 1.000 | 0.376 | 0.437 | 1.000 | 1.000 | 1.000 | 0.048 |  |  |  |  |
| Color 13 | | 1.000 | 1.000 | 1.000 | 1.000 | 1.000 | 1.000 | 1.000 | 1.000 | 1.000 | 1.000 | 1.000 | 1.000 |  |  |  |
| Color 14 | | 1.000 | 1.000 | 0.648 | 1.000 | 1.000 | 0.208 | 0.432 | 1.000 | 1.000 | 1.000 | 0.023 | 1.000 | 1.000 |  |  |
| Color 15 | | 1.000 | 1.000 | 1.000 | 1.000 | 1.000 | 1.000 | 1.000 | 1.000 | 1.000 | 1.000 | 1.000 | 1.000 | 1.000 | 1.000 |  |
|  | | < 0.001 | < 0.001 | < 0.001 | < 0.001 | < 0.001 | < 0.001 | < 0.001 | < 0.001 | < 0.001 | < 0.001 | < 0.001 | 0.003 | < 0.001 | 0.006 | < 0.001 |
|  |  |  |  |  |  |  |  |  |  |  |  |  |  |  |  |  |
| Mean | -0.136 | 0.117 | 0.201 | 0.220 | 0.193 | 0.179 | 0.234 | 0.235 | 0.131 | 0.190 | 0.159 | 0.259 | 0.086 | 0.166 | 0.080 | 0.137 |
| SEM | 0.037 | 0.038 | 0.039 | 0.038 | 0.037 | 0.038 | 0.038 | 0.041 | 0.038 | 0.039 | 0.039 | 0.039 | 0.038 | 0.039 | 0.036 | 0.038 |

Table S2 cont.

F_middle frequency (15, 220)_ = 7.82, p < 0.001

| Power VW  Middle Frequency  Corrected p values | | Color 1 | Color 2 | Color 3 | Color 4 | Color 5 | Color 6 | Color 7 | Color 8 | Color 9 | Color 10 | Color 11 | Color 12 | Color 13 | Color 14 | Color 15 |
| --- | --- | --- | --- | --- | --- | --- | --- | --- | --- | --- | --- | --- | --- | --- | --- | --- |
| Color 1 | |  |  |  |  |  |  |  |  |  |  |  |  |  |  |  |
| Color 2 | | 1.000 |  |  |  |  |  |  |  |  |  |  |  |  |  |  |
| Color 3 | | 1.000 | 1.000 |  |  |  |  |  |  |  |  |  |  |  |  |  |
| Color 4 | | 1.000 | 1.000 | 1.000 |  |  |  |  |  |  |  |  |  |  |  |  |
| Color 5 | | 1.000 | 1.000 | 1.000 | 1.000 |  |  |  |  |  |  |  |  |  |  |  |
| Color 6 | | 0.226 | 1.000 | 1.000 | 1.000 | 1.000 |  |  |  |  |  |  |  |  |  |  |
| Color 7 | | 0.057 | 1.000 | 1.000 | 1.000 | 1.000 | 1.000 |  |  |  |  |  |  |  |  |  |
| Color 8 | | 1.000 | 1.000 | 1.000 | 1.000 | 1.000 | 1.000 | 0.932 |  |  |  |  |  |  |  |  |
| Color 9 | | 1.000 | 1.000 | 1.000 | 1.000 | 1.000 | 1.000 | 1.000 | 1.000 |  |  |  |  |  |  |  |
| Color 10 | | 1.000 | 1.000 | 1.000 | 1.000 | 1.000 | 0.326 | 0.111 | 1.000 | 1.000 |  |  |  |  |  |  |
| Color 11 | | 1.000 | 1.000 | 1.000 | 1.000 | 1.000 | 1.000 | 1.000 | 1.000 | 1.000 | 1.000 |  |  |  |  |  |
| Color 12 | | 1.000 | 1.000 | 1.000 | 1.000 | 1.000 | 1.000 | 0.217 | 1.000 | 1.000 | 1.000 | 1.000 |  |  |  |  |
| Color 13 | | 1.000 | 1.000 | 1.000 | 1.000 | 1.000 | 1.000 | 1.000 | 1.000 | 1.000 | 1.000 | 1.000 | 1.000 |  |  |  |
| Color 14 | | 1.000 | 0.125 | 0.278 | 1.000 | 0.054 | 0.003 | < 0.001 | 1.000 | 0.052 | 1.000 | 0.057 | 1.000 | 0.236 |  |  |
| Color 15 | | 1.000 | 1.000 | 1.000 | 1.000 | 1.000 | 1.000 | 1.000 | 1.000 | 1.000 | 1.000 | 1.000 | 1.000 | 1.000 | 1.000 |  |
|  | | < 0.001 | < 0.001 | < 0.001 | < 0.001 | < 0.001 | < 0.001 | < 0.001 | < 0.001 | < 0.001 | < 0.001 | < 0.001 | < 0.001 | < 0.001 | 0.006 | < 0.001 |
|  |  |  |  |  |  |  |  |  |  |  |  |  |  |  |  |  |
| Mean | -0.146 | 0.096 | 0.185 | 0.180 | 0.156 | 0.194 | 0.232 | 0.249 | 0.136 | 0.199 | 0.107 | 0.192 | 0.115 | 0.176 | 0.046 | 0.155 |
| SEM | 0.032 | 0.033 | 0.033 | 0.033 | 0.031 | 0.032 | 0.033 | 0.033 | 0.033 | 0.033 | 0.033 | 0.034 | 0.033 | 0.032 | 0.031 | 0.033 |

Table S2 cont.

F_high frequency (15, 220)_ = 6.58, p < 0.001

| Power VW  High Frequency  Corrected p values | | Color 1 | Color 2 | Color 3 | Color 4 | Color 5 | Color 6 | Color 7 | Color 8 | Color 9 | Color 10 | Color 11 | Color 12 | Color 13 | Color 14 | Color 15 |
| --- | --- | --- | --- | --- | --- | --- | --- | --- | --- | --- | --- | --- | --- | --- | --- | --- |
| Color 1 | |  |  |  |  |  |  |  |  |  |  |  |  |  |  |  |
| Color 2 | | 1.000 |  |  |  |  |  |  |  |  |  |  |  |  |  |  |
| Color 3 | | 1.000 | 1.000 |  |  |  |  |  |  |  |  |  |  |  |  |  |
| Color 4 | | 1.000 | 1.000 | 1.000 |  |  |  |  |  |  |  |  |  |  |  |  |
| Color 5 | | 1.000 | 1.000 | 1.000 | 1.000 |  |  |  |  |  |  |  |  |  |  |  |
| Color 6 | | 1.000 | 1.000 | 1.000 | 1.000 | 1.000 |  |  |  |  |  |  |  |  |  |  |
| Color 7 | | 1.000 | 1.000 | 1.000 | 1.000 | 1.000 | 1.000 |  |  |  |  |  |  |  |  |  |
| Color 8 | | 1.000 | 1.000 | 1.000 | 1.000 | 1.000 | 1.000 | 0.914 |  |  |  |  |  |  |  |  |
| Color 9 | | 1.000 | 1.000 | 1.000 | 1.000 | 1.000 | 1.000 | 1.000 | 1.000 |  |  |  |  |  |  |  |
| Color 10 | | 1.000 | 1.000 | 1.000 | 1.000 | 1.000 | 1.000 | 1.000 | 1.000 | 1.000 |  |  |  |  |  |  |
| Color 11 | | 1.000 | 1.000 | 1.000 | 1.000 | 1.000 | 1.000 | 1.000 | 1.000 | 1.000 | 1.000 |  |  |  |  |  |
| Color 12 | | 1.000 | 0.661 | 1.000 | 1.000 | 0.456 | 1.000 | 0.190 | 1.000 | 0.495 | 1.000 | 0.711 |  |  |  |  |
| Color 13 | | 1.000 | 1.000 | 1.000 | 1.000 | 1.000 | 1.000 | 1.000 | 1.000 | 1.000 | 1.000 | 1.000 | 1.000 |  |  |  |
| Color 14 | | 1.000 | 1.000 | 1.000 | 1.000 | 1.000 | 1.000 | 0.863 | 1.000 | 1.000 | 1.000 | 1.000 | 1.000 | 1.000 |  |  |
| Color 15 | | 1.000 | 1.000 | 1.000 | 1.000 | 1.000 | 1.000 | 1.000 | 1.000 | 1.000 | 1.000 | 1.000 | 1.000 | 1.000 | 1.000 |  |
|  | | < 0.001 | < 0.001 | < 0.001 | < 0.001 | < 0.001 | < 0.001 | < 0.001 | < 0.001 | < 0.001 | < 0.001 | < 0.001 | < 0.001 | < 0.001 | < 0.001 | < 0.001 |
|  |  |  |  |  |  |  |  |  |  |  |  |  |  |  |  |  |
| Mean | -0.141 | 0.088 | 0.163 | 0.133 | 0.108 | 0.163 | 0.148 | 0.175 | 0.083 | 0.164 | 0.082 | 0.164 | 0.059 | 0.111 | 0.074 | 0.118 |
| SEM | 0.029 | 0.028 | 0.027 | 0.029 | 0.025 | 0.026 | 0.027 | 0.028 | 0.026 | 0.028 | 0.028 | 0.028 | 0.027 | 0.028 | 0.028 | 0.029 |

Table S2 Detailed statistical results of z-scored power. Compared between 15 colors. Done by one-way repeated measures ANOVA, Bonferroni-adjusted significance tests for pairwise comparisons. *p* < 0.05 are labeled by red.

Table S3 (A). One-way ANOVA; stimulated by 15 colors

|  | F_low frequency (3, 8340)_ = 7.68, p < 0.001 | | | | | | |
| --- | --- | --- | --- | --- | --- | --- | --- |
| Power  Low Frequency  Corrected p values | | NCL | ROT | ENTO | Mean | SEM |  |
| NCL | |  |  |  | 0.223 | 0.012 |  |
| ROT | | 0.001 |  |  | 0.157 | 0.012 |  |
| ENTO | | 1.000 | < 0.001 |  | 0.232 | 0.013 |  |
| VW | | 1.000 | 0.186 | 0.356 | 0.197 | 0.014 |  |
|  | |  |  |  |  |  |  |
| F_middle frequency (3, 8340)_ = 14.65, p < 0.001 | | | | |  |  |  |
| Power  Middle Frequency  Corrected p values | | NCL | ROT | ENTO | Mean | SEM |  |
| NCL | |  |  |  | 0.248 | 0.012 |  |
| ROT | | < 0.001 |  |  | 0.183 | 0.011 |  |
| ENTO | | 0.989 | < 0.001 |  | 0.271 | 0.012 |  |
| VW | | 0.001 | 1.000 | < 0.001 | 0.183 | 0.012 |  |
| F_high frequency (3, 8340)_ = 22.41, p < 0.001 | | | | |  |  |  |
| Power  High Frequency  Corrected p values | | NCL | ROT | ENTO | Mean | SEM |  |
| NCL | |  |  |  | 0.218 | 0.011 |  |
| ROT | | < 0.001 |  |  | 0.152 | 0.010 |  |
| ENTO | | 0.462 | < 0.001 |  | 0.244 | 0.011 |  |
| VW | | < 0.001 | 1.000 | < 0.001 | 0.140 | 0.010 |  |

Table S3 (B). One-way ANOVA; stimulate by Rainbow colors

| F_low frequency (3, 4196)_ = 11.49, p < 0.001 | | | | | | | | |  |  |
| --- | --- | --- | --- | --- | --- | --- | --- | --- | --- | --- |
| Power  Low Frequency  Corrected p values | | | | | | NCL | ROT | ENTO | Mean | SEM |
| NCL | | | | | |  |  |  | 0.169 | 0.018 |
| ROT | | | | | | 1.000 |  |  | 0.152 | 0.016 |
| ENTO | | | | | | 0.187 | 0.026 |  | 0.221 | 0.018 |
| VW | | | | | | 0.001 | 0.009 | < 0.001 | 0.069 | 0.019 |
| F_middle frequency (3, 8340)_ = 12.48, p < 0.001 | | | | | | | | |  |  |
| Power  Middle Frequency  Corrected p values | | | | | | NCL | ROT | ENTO | Mean | SEM |
| NCL | | | | | |  |  |  | 0.177 | 0.017 |
| ROT | | | | | | 1.000 |  |  | 0.155 | 0.014 |
| ENTO | | | | | | 0.008 | < 0.001 |  | 0.249 | 0.017 |
| VW | | | | | | 0.021 | 0.261 | < 0.001 | 0.107 | 0.017 |
| F_low frequency (3, 8340)_ =14.05, p < 0.001 | | | | | | | | |  |  |
| Power  High Frequency  Corrected p values | | | | | | NCL | ROT | ENTO | Mean | SEM |
| NCL | | | | | |  |  |  | 0.193 | 0.016 |
| ROT | | | | | | 0.028 |  |  | 0.133 | 0.013 |
| ENTO | | | | | | 1.000 | < 0.001 |  | 0.218 | 0.016 |
| VW | | | | | | < 0.001 | 0.208 | < 0.001 | 0.086 | 0.016 |
|  |  |  |  |  |  |  |  |  |  |  |

Table S3 (C). One-way repeated measures ANOVA; stimulated by Rainbow colors

|  |  |  |  |  |  |  |  |
| --- | --- | --- | --- | --- | --- | --- | --- |
| F_low frequency (3, 8340)_ = 7.68, p < 0.001 | | | |  |  |  |  |
| Power  Low Frequency  Corrected p values | | NCL | ROT | | ENTO | Mean | SEM |
| NCL | |  |  | |  | 0.097 | 0.018 |
| ROT | | 1.000 |  | |  | 0.082 | 0.018 |
| ENTO | | < 0.001 | < 0.001 | |  | 0.171 | 0.020 |
| VW | | 0.401 | 1.000 | | < 0.001 | 0.069 | 0.019 |
| F_middle frequency (3, 837)_ = 15.96, p < 0.001 | | | | | |  |  |
| Power  Middle Frequency  Corrected p values | | NCL | ROT | | ENTO | Mean | SEM |
| NCL | |  |  | |  | 0.107 | 0.017 |
| ROT | | 0.881 |  | |  | 0.085 | 0.014 |
| ENTO | | < 0.001 | < 0.001 | |  | 0.198 | 0.019 |
| VW | | 1.000 | 0.904 | | < 0.001 | 0.107 | 0.017 |
| F_high frequency (3, 837)_ = 11.66, p < 0.001 | | | | | |  |  |
| Power  High Frequency  Corrected p values | | NCL | ROT | | ENTO | Mean | SEM |
| NCL | |  |  | |  | 0.130 | 0.016 |
| ROT | | < 0.001 |  | |  | 0.060 | 0.013 |
| ENTO | | 0.110 | < 0.001 | |  | 0.163 | 0.018 |
| VW | | 0.027 | 0.524 | | < 0.001 | 0.086 | 0.016 |

Table S3 (D). One-way repeated measures ANOVA; stimulated by blue

| F_low frequency (3, 117)_ = 3.97, p = 0.010 | | | |  |  |
| --- | --- | --- | --- | --- | --- |
| Power  Low Frequency  Corrected p values | NCL | ROT | ENTO | Mean | SEM |
| NCL |  |  |  | 0.237 | 0.051 |
| ROT | 0.242 |  |  | 0.146 | 0.049 |
| ENTO | 0.610 | 0.015 |  | 0.299 | 0.055 |
| VW | 0.180 | 1.000 | 0.010 | 0.144 | 0.054 |
| F_middle frequency (3, 117)_ = 10.17, p < 0.001 | | | |  |  |
| Power  Middle Frequency  Corrected p values | NCL | ROT | ENTO | Mean | SEM |
| NCL |  |  |  | 0.264 | 0.044 |
| ROT | < 0.001 |  |  | 0.081 | 0.039 |
| ENTO | 0.684 | < 0.001 |  | 0.325 | 0.051 |
| VW | 1.000 | < 0.001 | 0.323 | 0.235 | 0.041 |
| F_high frequency (3, 117)_ = 7.89, p < 0.001 | | | |  |  |
| Power  High Frequency  Corrected p values | NCL | ROT | ENTO | Mean | SEM |
| NCL |  |  |  | 0.249 | 0.044 |
| ROT | < 0.001 |  |  | 0.062 | 0.038 |
| ENTO | 1.000 | < 0.001 |  | 0.279 | 0.047 |
| VW | 0.196 | 0.304 | 0.042 | 0.146 | 0.044 |

Table S3 Detailed statistical results of z-scored power when compared between four brain areas. (A). After pooling the power of 15 colors. Done by one-way ANOVA. (B). After pooling the power of Rainbow colors. Done by one-way ANOVA. (C) Done by one-way repeated measures ANOVA and some trails with missing values were excluded. (D). Analysis of the data from blue stimulation. Done by one-way repeated measures ANOVA and some trails with missing values were excluded. Bonferroni-adjusted significance tests for pairwise comparisons. *p* < 0.05 are labeled by red.

Table S4. One-way repeated measures ANOVA

F_low frequency (6, 154)_ = 7.23, p < 0.001

| Power NCL  Low Frequency  Corrected p values | Red | Orange | Yellow | Green | Blue | Indigo | Mean | SEM |  |
| --- | --- | --- | --- | --- | --- | --- | --- | --- | --- |
| Red |  |  |  |  |  |  | 0.089 | 0.043 |  |
| Orange | 1.000 |  |  |  |  |  | 0.110 | 0.045 |  |
| Yellow | 1.000 | 1.000 |  |  |  |  | 0.104 | 0.045 |  |
| Green | 1.000 | 1.000 | 1.000 |  |  |  | 0.145 | 0.045 |  |
| Blue | < 0.001 | < 0.001 | < 0.001 | 0.001 |  |  | 0.407 | 0.048 |  |
| Indigo | 1.000 | 1.000 | 1.000 | 1.000 | < 0.001 |  | 0.100 | 0.046 |  |
| Violet | 0.635 | 1.000 | 1.000 | 1.000 | 0.102 | 1.000 | 0.227 | 0.049 |  |
|  |  |  |  |  |  |  |  |  |  |
| F_middle frequency (6, 154)_ = 9.35, p < 0.001 | | | | | | |  |  |  |
| Power NCL  Middle Frequency  Corrected p values | Red | Orange | Yellow | Green | Blue | Indigo | Mean | SEM |  |
| Red |  |  |  |  |  |  | 0.154 | 0.043 |  |
| Orange | 1.000 |  |  |  |  |  | 0.060 | 0.041 |  |
| Yellow | 1.000 | 1.000 |  |  |  |  | 0.123 | 0.040 |  |
| Green | 1.000 | 1.000 | 1.000 |  |  |  | 0.161 | 0.044 |  |
| Blue | < 0.001 | < 0.001 | < 0.001 | < 0.001 |  |  | 0.435 | 0.045 |  |
| Indigo | 1.000 | 1.000 | 1.000 | 1.000 | < 0.001 |  | 0.098 | 0.042 |  |
| Violet | 1.000 | 0.154 | 1.000 | 1.000 | 0.003 | 1.000 | 0.210 | 0.045 |  |

F_high frequency (6, 154)_ = 7.67, p < 0.001

| Power NCL High Frequency  Corrected p values | Red | Orange | Yellow | Green | Blue | Indigo | Mean | SEM |
| --- | --- | --- | --- | --- | --- | --- | --- | --- |
| Red |  |  |  |  |  |  | 0.166 | 0.040 |
| Orange | 1.000 |  |  |  |  |  | 0.125 | 0.038 |
| Yellow | 1.000 | 1.000 |  |  |  |  | 0.102 | 0.039 |
| Green | 1.000 | 1.000 | 1.000 |  |  |  | 0.169 | 0.041 |
| Blue | < 0.001 | < 0.001 | < 0.001 | < 0.001 |  |  | 0.424 | 0.045 |
| Indigo | 1.000 | 1.000 | 1.000 | 1.000 | < 0.001 |  | 0.106 | 0.040 |
| Violet | 1.000 | 0.253 | 0.094 | 1.000 | 0.080 | 0.116 | 0.257 | 0.042 |

Table S4 cont.

F_low frequency (6, 154)_ = 2.14, p = 0.052

| Power ROT  Low Frequency  Corrected p values | Red | Orange | Yellow | Green | Blue | Indigo | Mean | SEM |  |  |  |  |  |  |
| --- | --- | --- | --- | --- | --- | --- | --- | --- | --- | --- | --- | --- | --- | --- |
| Red |  |  |  |  |  |  | 0.142 | 0.043 |  |  |  |  |  |  |
| Orange | 1.000 |  |  |  |  |  | 0.101 | 0.040 |  |  |  |  |  |  |
| Yellow | 1.000 | 1.000 |  |  |  |  | 0.092 | 0.044 |  |  |  |  |  |  |
| Green | 1.000 | 1.000 | 1.000 |  |  |  | 0.158 | 0.043 |  |  |  |  |  |  |
| Blue | 0.504 | 0.057 | 0.081 | 1.000 |  |  | 0.268 | 0.045 |  |  |  |  |  |  |
| Indigo | 1.000 | 1.000 | 1.000 | 1.000 | 0.187 |  | 0.119 | 0.040 |  |  |  |  |  |  |
| Violet | 1.000 | 1.000 | 1.000 | 1.000 | 1.000 | 1.000 | 0.184 | 0.044 |  |  |  |  |  |  |
|  |  |  |  |  |  |  |  |  |  |  |  |  |  |  |
| F_middle frequency (6, 154)_ = 3.19, p = 0.006 | | | | | | |  |  |  |  |  |  |  |  |
| Power ROT Middle Frequency  Corrected p values | Red | Orange | Yellow | Green | Blue | Indigo | Mean | SEM |  |  |  |  |  |  |
| Red |  |  |  |  |  |  | 0.191 | 0.035 |  |  |  |  |  |  |
| Orange | 1.000 |  |  |  |  |  | 0.183 | 0.034 |  |  |  |  |  |  |
| Yellow | 1.000 | 1.000 |  |  |  |  | 0.127 | 0.035 |  |  |  |  |  |  |
| Green | 0.048 | 0.100 | 1.000 |  |  |  | 0.049 | 0.035 |  |  |  |  |  |  |
| Blue | 1.000 | 1.000 | 0.385 | 0.002 |  |  | 0.245 | 0.041 |  |  |  |  |  |  |
| Indigo | 1.000 | 1.000 | 1.000 | 1.000 | 0.225 |  | 0.129 | 0.035 |  |  |  |  |  |  |
| Violet | 1.000 | 1.000 | 1.000 | 0.590 | 1.000 | 1.000 | 0.163 | 0.040 |  |  |  |  |  |  |
|  |  |  |  |  |  |  |  |  |  |  |  |  |  |  |
| F_high frequency (6, 154)_ = 1.72, p = 0.120 | | | | | | |  |  |  |  |  |  |  |  |
| Power ROT  High Frequency  Corrected p values | Red | Orange | Yellow | Green | Blue | Indigo | Mean | SEM |  |  |  |  |  |  |
| Red |  |  |  |  |  |  | 0.150 | 0.033 |  |  |  |  |  |  |
| Orange | 1.000 |  |  |  |  |  | 0.092 | 0.032 |  |  |  |  |  |  |
| Yellow | 1.000 | 1.000 |  |  |  |  | 0.147 | 0.032 |  |  |  |  |  |  |
| Green | 1.000 | 1.000 | 1.000 |  |  |  | 0.112 | 0.033 |  |  |  |  |  |  |
| Blue | 1.000 | 0.086 | 1.000 | 0.731 |  |  | 0.213 | 0.039 |  |  |  |  |  |  |
| Indigo | 1.000 | 1.000 | 1.000 | 1.000 | 0.783 |  | 0.122 | 0.034 |  |  |  |  |  |  |
| Violet | 1.000 | 1.000 | 1.000 | 1.000 | 0.384 | 1.000 | 0.098 | 0.033 |  |  |  |  |  |  |

Table S4 cont.

F_low frequency (6, 154)_ = 5.16, p < 0.001

| Power ENTO  Low Frequency  Corrected p values | Red | Orange | Yellow | Green | Blue | Indigo | Mean | SEM |
| --- | --- | --- | --- | --- | --- | --- | --- | --- |
| Red |  |  |  |  |  |  | 0.152 | 0.047 |
| Orange | 1.000 |  |  |  |  |  | 0.192 | 0.047 |
| Yellow | 1.000 | 1.000 |  |  |  |  | 0.142 | 0.044 |
| Green | 1.000 | 1.000 | 1.000 |  |  |  | 0.203 | 0.046 |
| Blue | < 0.001 | 0.001 | < 0.001 | 0.011 |  |  | 0.444 | 0.050 |
| Indigo | 1.000 | 1.000 | 1.000 | 1.000 | 0.001 |  | 0.157 | 0.049 |
| Violet | 1.000 | 1.000 | 1.000 | 1.000 | 0.115 | 1.000 | 0.258 | 0.050 |
|  |  |  |  |  |  |  |  |  |
| F_middle frequency (6, 154)_ = 6.07, p < 0.001 | | | | | | |  |  |
| Power ENTO Middle Frequency  Corrected p values | Red | Orange | Yellow | Green | Blue | Indigo | Mean | SEM |
| Red |  |  |  |  |  |  | 0.184 | 0.045 |
| Orange | 1.000 |  |  |  |  |  | 0.226 | 0.046 |
| Yellow | 1.000 | 1.000 |  |  |  |  | 0.195 | 0.043 |
| Green | 1.000 | 1.000 | 1.000 |  |  |  | 0.247 | 0.044 |
| Blue | < 0.001 | 0.002 | < 0.001 | 0.013 |  |  | 0.464 | 0.048 |
| Indigo | 1.000 | 1.000 | 1.000 | 1.000 | < 0.001 |  | 0.167 | 0.047 |
| Violet | 1.000 | 1.000 | 1.000 | 1.000 | 0.025 | 1.000 | 0.258 | 0.047 |
|  |  |  |  |  |  |  |  |  |
| F_high frequency (6, 154)_ = 6.84, p < 0.001 | | | | | | |  |  |
| Power ENTO High Frequency  Corrected p values | Red | Orange | Yellow | Green | Blue | Indigo | Mean | SEM |
| Red |  |  |  |  |  |  | 0.165 | 0.043 |
| Orange | 1.000 |  |  |  |  |  | 0.208 | 0.041 |
| Yellow | 1.000 | 1.000 |  |  |  |  | 0.147 | 0.040 |
| Green | 1.000 | 1.000 | 1.000 |  |  |  | 0.193 | 0.046 |
| Blue | < 0.001 | 0.001 | < 0.001 | 0.001 |  |  | 0.430 | 0.046 |
| Indigo | 1.000 | 1.000 | 1.000 | 1.000 | < 0.001 |  | 0.122 | 0.041 |
| Violet | 1.000 | 1.000 | 1.000 | 1.000 | 0.085 | 0.369 | 0.259 | 0.044 |

Table S4 cont.

F_low frequency (6, 114)_ = 1.56, p = 0.165

| Power VW  Low Frequency  Corrected p values | Red | Orange | Yellow | Green | Blue | Indigo | | Mean | | SEM | |  |  |  |  |  |  |
| --- | --- | --- | --- | --- | --- | --- | --- | --- | --- | --- | --- | --- | --- | --- | --- | --- | --- |
| Red |  |  |  |  |  |  | | -0.008 | | 0.050 | |  |  |  |  |  |  |
| Orange | 1.000 |  |  |  |  |  | | 0.062 | | 0.047 | |  |  |  |  |  |  |
| Yellow | 1.000 | 1.000 |  |  |  |  | | 0.084 | | 0.052 | |  |  |  |  |  |  |
| Green | 1.000 | 1.000 | 1.000 |  |  |  | | 0.005 | | 0.050 | |  |  |  |  |  |  |
| Blue | 0.788 | 1.000 | 1.000 | 1.000 |  |  | | 0.144 | | 0.054 | |  |  |  |  |  |  |
| Indigo | 1.000 | 1.000 | 1.000 | 1.000 | 1.000 |  | | 0.032 | | 0.048 | |  |  |  |  |  |  |
| Violet | 0.426 | 1.000 | 1.000 | 0.404 | 1.000 | 1.000 | | 0.162 | | 0.051 | |  |  |  |  |  |  |
|  |  |  |  |  |  |  | |  | |  | |  |  |  |  |  |  |
| F_middle frequency (6, 114)_ = 3.51, p = 0.003 | | | | | | |  | |  | |  | |  |  |  |  |  |
| Power VW  Middle Frequency  Corrected p values | Red | Orange | Yellow | Green | Blue | Indigo | | Mean | | SEM | |  |  |  |  |  |  |
| Red |  |  |  |  |  |  | | 0.076 | | 0.045 | |  |  |  |  |  |  |
| Orange | 1.000 |  |  |  |  |  | | 0.089 | | 0.044 | |  |  |  |  |  |  |
| Yellow | 1.000 | 1.000 |  |  |  |  | | 0.164 | | 0.042 | |  |  |  |  |  |  |
| Green | 1.000 | 1.000 | 0.805 |  |  |  | | 0.042 | | 0.045 | |  |  |  |  |  |  |
| Blue | 0.190 | 0.255 | 1.000 | 0.012 |  |  | | 0.235 | | 0.041 | |  |  |  |  |  |  |
| Indigo | 1.000 | 1.000 | 0.310 | 1.000 | 0.007 |  | | 0.021 | | 0.045 | |  |  |  |  |  |  |
| Violet | 1.000 | 1.000 | 1.000 | 1.000 | 0.978 | 1.000 | | 0.120 | | 0.050 | |  |  |  |  |  |  |
|  |  |  |  |  |  |  | |  | |  | |  |  |  |  |  |  |
| F_high frequency (6, 114)_ = 1.10, p = 0.05 | | | | | | |  | |  | |  | |  |  |  |  |  |
| Power VW  High Frequency  Corrected p values | Red | Orange | Yellow | Green | Blue | Indigo | | Mean | | SEM | |  |  |  |  |  |  |
| Red |  |  |  |  |  |  | | 0.061 | | 0.038 | |  |  |  |  |  |  |
| Orange | 1.000 |  |  |  |  |  | | 0.101 | | 0.041 | |  |  |  |  |  |  |
| Yellow | 1.000 | 1.000 |  |  |  |  | | 0.106 | | 0.039 | |  |  |  |  |  |  |
| Green | 1.000 | 1.000 | 1.000 |  |  |  | | 0.026 | | 0.042 | |  |  |  |  |  |  |
| Blue | 1.000 | 1.000 | 1.000 | 0.541 |  |  | | 0.146 | | 0.044 | |  |  |  |  |  |  |
| Indigo | 1.000 | 1.000 | 1.000 | 1.000 | 0.541 |  | | 0.070 | | 0.042 | |  |  |  |  |  |  |
| Violet | 1.000 | 1.000 | 1.000 | 1.000 | 1.000 | 1.000 | | 0.090 | | 0.044 | |  |  |  |  |  |  |

Table S4 Detailed statistical results of z-scored power. Compared between rainbow colors. Done by one-way repeated measures ANOVA, Bonferroni-adjusted significance tests for pairwise comparisons. *p* < 0.05 are labeled by red.

Table S5. One-way repeated measures ANOVA

F_low frequency (6, 154)_ = 3.18, p = 0.006

| WPLI ROT-NCL  Low Frequency  Corrected p values | Red | Orange | Yellow | Green | Blue | Indigo | Mean | SEM |
| --- | --- | --- | --- | --- | --- | --- | --- | --- |
| Red |  |  |  |  |  |  | 0.037 | 0.022 |
| Orange | 1.000 |  |  |  |  |  | 0.016 | 0.021 |
| Yellow | 1.000 | 1.000 |  |  |  |  | 0.062 | 0.021 |
| Green | 1.000 | 1.000 | 0.766 |  |  |  | 0.002 | 0.018 |
| Blue | 1.000 | 1.000 | 0.220 | 1.000 |  |  | -0.011 | 0.019 |
| Indigo | 1.000 | 1.000 | 1.000 | 0.370 | 0.108 |  | 0.062 | 0.021 |
| Violet | 0.352 | 1.000 | 0.029 | 1.000 | 1.000 | 0.014 | -0.028 | 0.018 |
|  |  |  |  |  |  |  |  |  |
| F_middle frequency (6, 154)_ = 1.89, p = 0.086 | | | | | | |  |  |
| WPLI ROT-NCL  Middle Frequency  Corrected p values | Red | Orange | Yellow | Green | Blue | Indigo | Mean | SEM |
| Red |  |  |  |  |  |  | 0.041 | 0.028 |
| Orange | 1.000 |  |  |  |  |  | -0.005 | 0.029 |
| Yellow | 1.000 | 0.198 |  |  |  |  | 0.092 | 0.030 |
| Green | 1.000 | 1.000 | 0.335 |  |  |  | -0.007 | 0.027 |
| Blue | 1.000 | 1.000 | 0.290 | 1.000 |  |  | -0.007 | 0.027 |
| Indigo | 1.000 | 1.000 | 1.000 | 1.000 | 1.000 |  | 0.040 | 0.029 |
| Violet | 1.000 | 1.000 | 0.180 | 1.000 | 1.000 | 1.000 | -0.013 | 0.026 |
|  |  |  |  |  |  |  |  |  |
| F_high frequency (6, 154)_ = 1.01, p = 0.420 | | | | | | |  |  |
| WPLI ROT-NCL  High Frequency  Corrected p values | Red | Orange | Yellow | Green | Blue | Indigo | Mean | SEM |
| Red |  |  |  |  |  |  | -0.005 | 0.026 |
| Orange | 1.000 |  |  |  |  |  | 0.002 | 0.028 |
| Yellow | 1.000 | 1.000 |  |  |  |  | 0.036 | 0.024 |
| Green | 1.000 | 1.000 | 1.000 |  |  |  | -0.031 | 0.027 |
| Blue | 1.000 | 1.000 | 1.000 | 1.000 |  |  | 0.027 | 0.026 |
| Indigo | 1.000 | 1.000 | 1.000 | 0.715 | 1.000 |  | 0.046 | 0.025 |
| Violet | 1.000 | 1.000 | 1.000 | 1.000 | 1.000 | 1.000 | 0.006 | 0.024 |

Table S5 cont.

F_low frequency (6, 154)_ = 1.55, p = 0.167

| WPLI ENTO-NCL  Low Frequency  Corrected p values | Red | Orange | Yellow | Green | Blue | Indigo | Mean | SEM |
| --- | --- | --- | --- | --- | --- | --- | --- | --- |
| Red |  |  |  |  |  |  | 0.045 | 0.017 |
| Orange | 1.000 |  |  |  |  |  | 0.029 | 0.017 |
| Yellow | 1.000 | 1.000 |  |  |  |  | 0.023 | 0.015 |
| Green | 1.000 | 1.000 | 1.000 |  |  |  | 0.008 | 0.015 |
| Blue | 1.000 | 1.000 | 0.566 | 0.078 |  |  | 0.072 | 0.017 |
| Indigo | 1.000 | 1.000 | 1.000 | 1.000 | 1.000 |  | 0.034 | 0.017 |
| Violet | 1.000 | 1.000 | 1.000 | 1.000 | 1.000 | 1.000 | 0.036 | 0.017 |
|  |  |  |  |  |  |  |  |  |
| F_middle frequency (6, 154)_ = 0.80, p = 0.569 | | | | | | |  |  |
| WPLI ENTO-NCL  Middle Frequency  Corrected p values | Red | Orange | Yellow | Green | Blue | Indigo | Mean | SEM |
| Red |  |  |  |  |  |  | 0.075 | 0.028 |
| Orange | 1.000 |  |  |  |  |  | 0.063 | 0.029 |
| Yellow | 1.000 | 1.000 |  |  |  |  | 0.032 | 0.028 |
| Green | 1.000 | 1.000 | 1.000 |  |  |  | 0.055 | 0.027 |
| Blue | 1.000 | 1.000 | 1.000 | 1.000 |  |  | 0.099 | 0.029 |
| Indigo | 1.000 | 1.000 | 1.000 | 1.000 | 1.000 |  | 0.055 | 0.030 |
| Violet | 1.000 | 1.000 | 1.000 | 1.000 | 1.000 | 1.000 | 0.098 | 0.028 |
|  |  |  |  |  |  |  |  |  |
| F_high frequency (6, 154)_ = 1.21, p = 0.303 | | | | | | |  |  |
| WPLI ENTO-NCL  High Frequency  Corrected p values | Red | Orange | Yellow | Green | Blue | Indigo | Mean | SEM |
| Red |  |  |  |  |  |  | 0.081 | 0.026 |
| Orange | 1.000 |  |  |  |  |  | 0.053 | 0.028 |
| Yellow | 1.000 | 1.000 |  |  |  |  | 0.025 | 0.026 |
| Green | 1.000 | 1.000 | 1.000 |  |  |  | 0.088 | 0.030 |
| Blue | 1.000 | 1.000 | 0.222 | 1.000 |  |  | 0.122 | 0.029 |
| Indigo | 1.000 | 1.000 | 1.000 | 1.000 | 1.000 |  | 0.075 | 0.027 |
| Violet | 1.000 | 1.000 | 1.000 | 1.000 | 1.000 | 1.000 | 0.064 | 0.027 |

Table S5 cont.

F_low frequency (6, 114)_ = 1.91, p = 0.086

| WPLI VW-NCL  Low Frequency  Corrected p values | Red | Orange | Yellow | Green | Blue | Indigo | Mean | SEM |
| --- | --- | --- | --- | --- | --- | --- | --- | --- |
| Red |  |  |  |  |  |  | 0.038 | 0.025 |
| Orange | 0.574 |  |  |  |  |  | -0.034 | 0.023 |
| Yellow | 1.000 | 1.000 |  |  |  |  | 0.029 | 0.023 |
| Green | 1.000 | 1.000 | 1.000 |  |  |  | -0.009 | 0.024 |
| Blue | 1.000 | 1.000 | 1.000 | 1.000 |  |  | -0.014 | 0.024 |
| Indigo | 1.000 | 0.443 | 1.000 | 1.000 | 1.000 |  | 0.053 | 0.025 |
| Violet | 1.000 | 1.000 | 1.000 | 1.000 | 1.000 | 0.485 | -0.036 | 0.028 |
|  |  |  |  |  |  |  |  |  |
| F_middle frequency (6, 114)_ = 1.00, p = 0.427 | | | | | | |  |  |
| WPLI VW-NCL  Middle Frequency  Corrected p values | Red | Orange | Yellow | Green | Blue | Indigo | Mean | SEM |
| Red |  |  |  |  |  |  | -0.023 | 0.035 |
| Orange | 1.000 |  |  |  |  |  | -0.024 | 0.030 |
| Yellow | 1.000 | 1.000 |  |  |  |  | 0.048 | 0.035 |
| Green | 1.000 | 1.000 | 1.000 |  |  |  | 0.016 | 0.031 |
| Blue | 1.000 | 1.000 | 0.569 | 1.000 |  |  | -0.059 | 0.031 |
| Indigo | 1.000 | 1.000 | 1.000 | 1.000 | 1.000 |  | -0.032 | 0.035 |
| Violet | 1.000 | 1.000 | 1.000 | 1.000 | 1.000 | 1.000 | -0.049 | 0.037 |
|  |  |  |  |  |  |  |  |  |
| F_high frequency (6, 154)_ = 0.77, p = 0.598 | | | | | | |  |  |
| WPLI VW-NCL  High Frequency  Corrected p values | Red | Orange | Yellow | Green | Blue | Indigo | Mean | SEM |
| Red |  |  |  |  |  |  | -0.013 | 0.032 |
| Orange | 1.000 |  |  |  |  |  | -0.028 | 0.035 |
| Yellow | 1.000 | 1.000 |  |  |  |  | -0.020 | 0.033 |
| Green | 1.000 | 1.000 | 1.000 |  |  |  | -0.008 | 0.035 |
| Blue | 1.000 | 1.000 | 1.000 | 1.000 |  |  | -0.083 | 0.031 |
| Indigo | 1.000 | 1.000 | 1.000 | 1.000 | 1.000 |  | -0.064 | 0.032 |
| Violet | 1.000 | 1.000 | 1.000 | 1.000 | 1.000 | 1.000 | -0.031 | 0.034 |

Table S5 Detailed statistical results of WPLI. Compared between rainbow colors. Done by one-way repeated measures ANOVA, Bonferroni-adjusted significance tests for pairwise comparisons. *p* < 0.05 are labeled by red.

Table S6 (A). One-way ANOVA; stimulated by Rainbow colors

| F_low frequency (2, 3077)_ = 4.01, p = 0.018 | | |  |  |
| --- | --- | --- | --- | --- |
| WPLI Low Frequency  Corrected p values | ROT-NCL | ENTO-NCL | Mean | SEM |
| ROT-NCL |  |  | 0.020 | 0.008 |
| ENTO-NCL | 0.407 |  | 0.035 | 0.006 |
| VW-NCL | 0.447 | 0.014 | 0.004 | 0.009 |
|  |  |  |  |  |
| F_middle frequency (2, 3077)_ = 13.90, p < 0.001 | | |  |  |
| WPLI  Middle Frequency  Corrected p values | ROT-NCL | ENTO-NCL | Mean | SEM |
| ROT-NCL |  |  | 0.020 | 0.011 |
| ENTO-NCL | 0.005 |  | 0.068 | 0.011 |
| VW-NCL | 0.066 | < 0.001 | -0.018 | 0.013 |
|  |  |  |  |  |
|  |  |  |  |  |
| F_high frequency (2, 3077)_ = 24.11, p < 0.001 | | |  |  |
| WPLI  High Frequency  Corrected p values | ROT-NCL | ENTO-NCL | Mean | SEM |
| ROT-NCL |  |  | 0.011 | 0.010 |
| ENTO-NCL | < 0.001 |  | 0.073 | 0.010 |
| VW-NCL | 0.009 | < 0.001 | -0.035 | 0.012 |

Table S6 (B). One-way repeated measures ANOVA; stimulated by Rainbow colors

|  |  | | F_low frequency (2, 838)_ = 2.64, p = 0.072 | | | | |
| --- | --- | --- | --- | --- | --- | --- | --- |
| WPLI Low Frequency  Corrected p values | | ROT-NCL | | ENTO-NCL | Mean | SEM |  |
| ROT-NCL | |  | |  | -0.010 | 0.008 |  |
| ENTO-NCL | | 0.098 | |  | 0.011 | 0.007 |  |
| VW-NCL | | 0.437 | | 1.000 | 0.004 | 0.009 |  |
|  | |  | |  |  |  |  |
|  |  | | F_middle frequency (2, 838)_ = 3.98, p = 0.019 | | | | |
| WPLI  Middle Frequency  Corrected p values | | ROT-NCL | | ENTO-NCL | Mean | SEM |  |
| ROT-NCL | |  | |  | -0.006 | 0.011 |  |
| ENTO-NCL | | 0.064 | |  | 0.030 | 0.012 |  |
| VW-NCL | | 1.000 | | 0.020 | -0.018 | 0.013 |  |
|  | |  | |  |  |  |  |
|  | |  | |  |  |  |  |
|  |  | | F_high frequency (2, 838)_ = 7.31, p = 0.001 | | | | |
| WPLI  High Frequency  Corrected p values | | ROT-NCL | | ENTO-NCL | Mean | SEM |  |
| ROT-NCL | |  | |  | -0.014 | 0.011 |  |
| ENTO-NCL | | 0.012 | |  | 0.032 | 0.011 |  |
| VW-NCL | | 0.376 | | 0.000 | -0.035 | 0.012 |  |

Table S6 (C). One-way repeated measures ANOVA; stimulated by blue

| F_low frequency (2, 118)_ = 3.33, p = 0.039 | | |  |  |
| --- | --- | --- | --- | --- |
| WPLI Low Frequency  Corrected p values | ROT-NCL | ENTO-NCL | Mean | SEM |
| ROT-NCL |  |  | -0.034 | 0.020 |
| ENTO-NCL | 0.034 |  | 0.032 | 0.017 |
| VW-NCL | 1.000 | 0.392 | -0.014 | 0.024 |
|  |  |  |  |  |
| F_middle frequency (2, 118)_ = 2.50, p = 0.086 | | |  |  |
| WPLI  Middle Frequency  Corrected p values | ROT-NCL | ENTO-NCL | Mean | SEM |
| ROT-NCL |  |  | -0.005 | 0.030 |
| ENTO-NCL | 0.871 |  | 0.037 | 0.030 |
| VW-NCL | 0.446 | 0.085 | -0.059 | 0.031 |
|  |  |  |  |  |
|  |  |  |  |  |
| F_high frequency (2, 118)_ = 6.15, p = 0.030 | | |  |  |
| WPLI  High Frequency  Corrected p values | ROT-NCL | ENTO-NCL | Mean | SEM |
| ROT-NCL |  |  | 0.013 | 0.031 |
| ENTO-NCL | 1.000 |  | 0.052 | 0.029 |
| VW-NCL | 0.017 | 0.010 | -0.083 | 0.031 |

Table S6 Detailed statistical results of WPLI when compared between ROT-NCL, ENTO-NCL, VW-NCL. (A). After pooling the power of rainbow colors. Done by one-way ANOVA. (B) Done by one-way repeated measures ANOVA and some trails with missing values were excluded. (C). Analysis of the data from blue stimulation. Done by one-way repeated measures ANOVA and some trails with missing values were excluded. Bonferroni-adjusted significance tests for pairwise comparisons. *p* < 0.05 are labeled by red.

Table S7 (A). One-way ANOVA; stimulated by Rainbow colors

| F_(2,6157)_ = 460.28, p < 0.001 | | |  | | | | |  | | |  |  |  |
| --- | --- | --- | --- | --- | --- | --- | --- | --- | --- | --- | --- | --- | --- |
| G.C.  Corrected p values | ROT to NCL | ENTO to NCL | Mean | | | | | SEM | | |  |  |  |
| ROT to NCL |  |  | 9.70E-03 | | | | | 1.29E-04 | | |  |  |  |
| ENTO to NCL | <0.001 |  | 1.94E-02 | | | | | 3.13E-04 | | |  |  |  |
| VW to NCL | <0.001 | <0.001 | 1.340E-02 | | | | | 2.20E-04 | | |  |  |  |
|  |  |  | |  | |  | | |  | | | |  |
| F_(2,6157)_ = 396.53, p < 0.001 | | | | |  | |  | | |  | | | |
| G.C.  Corrected p values | NCL to ROT | NCL to ENTO | Mean | | | | | SEM | | |  |  |  |
| NCL to ROT |  |  | 1.07E-02 | | | | | 1.47E-04 | | |  |  |  |
| NCL to ENTO | <0.001 |  | 1.91E-02 | | | | | 2.92E-04 | | |  |  |  |
| NCL to VW | <0.001 | <0.001 | 1.25E-02 | | | | | 2.19E-04 | | |  |  |  |

Table S7 (B). One-way repeated measures ANVOA; stimulated by Rainbow colors

| F_(2,838)_ = 233.84, p < 0.001 | | |  |  |
| --- | --- | --- | --- | --- |
| G.C.  Corrected p values | ROT to NCL | ENTO to NCL | Mean | SEM |
| ROT to NCL |  |  | 8.42E-03 | 1.40E-04 |
| ENTO to NCL | <0.001 |  | 1.56E-02 | 4.16E-04 |
| VW to NCL | <0.001 | <0.001 | 1.25E-02 | 2.78E-04 |
|  |  |  |  |  |
| F_(2,838)_ = 69.94, p < 0.001 | | |  |  |
| G.C.  Corrected p values | NCL to ROT | NCL to ENTO | Mean | SEM |
| NCL to ROT |  |  | 1.09E-02 | 2.40E-04 |
| NCL to ENTO | <0.001 |  | 1.61E-02 | 3.99E-04 |
| NCL to VW | 0.001 | <0.001 | 1.19E-02 | 2.90E-04 |

Table S7 (C). One-way repeated measures ANOVA; stimulated by blue

| F_(2,118)_ = 26.23, p < 0.001 | | |  |  |
| --- | --- | --- | --- | --- |
| G.C.  Corrected p values | ROT to NCL | ENTO to NCL | Mean | SEM |
| ROT to NCL |  |  | 8.54E-03 | 3.66E-04 |
| ENTO to NCL | <0.001 |  | 1.55E-02 | 1.19E-03 |
| VW to NCL | <0.001 | 0.008 | 1.10E-02 | 5.73E-04 |
|  |  |  |  |  |
| F_(2,118)_ = 12.06, p < 0.001 | | |  |  |
| G.C.  Corrected p values | NCL to ROT | NCL to ENTO | Mean | SEM |
| NCL to ROT |  |  | 1.13E-02 | 6.56E-04 |
| NCL to ENTO | 0.001 |  | 1.57E-02 | 9.33E-04 |
| NCL to VW | 1.000 | <0.001 | 1.06E-02 | 5.90E-04 |

Table S7 (D). One-way repeated measures ANOVA; stimulated by Rainbow colors

| F _(1,1119)_ = 114.20 | | | | |
| --- | --- | --- | --- | --- |
| G.C.  p values | ROT to ENTO | Mean | SEM |  |
| ENTO to ROT | <0.001 | 8.30E-03 | 1.40E-04 |  |
| ROT to ENTO |  | 1.06E-02 | 1.79E-04 |  |
| Table S7 (E). One-way repeated measures ANOVA; stimulated by Blue  F _(1,159)_ = 11.23 | | | | |
| G.C.  p values | ROT to ENTO | Mean | SEM |  |
| ENTO to ROT | <0.001 | 7.80E-03 | 3.57E-04 |  |
| ROT to ENTO |  | 9.60E-03 | 4.21E-04 |  |

Table S7 Detailed statistical results of Granger causality (G.C.) when compared between ROT⬄NCL, ENTO⬄NCL, VW⬄NCL. (A). After pooling the power of rainbow colors. Done by one-way ANOVA. (B) Done by one-way repeated measures ANOVA and some trails with missing values were excluded. (C). Analysis of the data from blue stimulation. Done by one-way repeated measures ANOVA and some trails with missing values were excluded. (D). G.C. values of ENTO to ROT and ROT to ENTO after being stimulated by rainbow colors (E). G.C. values of ENTO to ROT and ROT to ENTO after being stimulated by blue. Bonferroni-adjusted significance tests for pairwise comparisons. *p* < 0.05 are labeled by red.

Table S8 (A). Paired *t*-test

| G.C.  ROT to NCL vs NCL to ROT | p values | t _(159)_= |
| --- | --- | --- |
| Red | 0.147 | -1.456 |
| Orange | 0.003 | -3.047 |
| Yellow | 0.016 | -2.441 |
| Green | 0.110 | -1.609 |
| Blue | 0.748 | -0.322 |
| Indigo | 0.003 | -2.992 |
| Violet | 0.194 | -1.303 |

Table S8 (B.)

| G.C.  ENTO to NCL vs NCL to ENTO | p values | t _(159)_= |
| --- | --- | --- |
| Red | 0.537 | 0.618 |
| Orange | 0.338 | 0.960 |
| Yellow | 0.957 | 0.053 |
| Green | 0.069 | 1.829 |
| Blue | <0.001 | 4.291 |
| Indigo | 0.017 | 2.420 |
| Violet | 0.002 | 3.217 |

Table S8 (C.)

| G.C.  VW to NCL vs NCL to VW | p values | t _(119)_= |
| --- | --- | --- |
| Red | 0.521 | 0.644 |
| Orange | 0.197 | 1.297 |
| Yellow | 0.792 | 0.264 |
| Green | 0.592 | 0.538 |
| Blue | 0.549 | 0.601 |
| Indigo | 0.077 | 1.787 |
| Violet | 0.624 | 0.492 |

Table S8 Granger causality directional analysis after stimulating by rainbow colors. (A) ROT to NCL vs NCL to ROT. (B) ENTO to NCL vs NCL to ENTO. (C) VW to NCL vs NCL to VW. Paired *t*-test. P < 0.05 are labeled by red.
